# Supplementary material for: Strategies selection for building e-commerce platforms for agricultural wholesale markets: A tripartite evolutionary game perspective
Source: PLoS One. 2024 Jan 25;19(1):e0297360. doi: 10.1371/journal.pone.0297360 (PMC10810498; doi:10.1371/journal.pone.0297360)
Supplement: S1 File — (DOCX) [file pone.0297360.s001.docx]

Figure 1：

clc,clear;

figure(11);

b=30;Cl=45;Ct=35;w2=20;I=30;T1=15;T2=10;A=30;Up=5;u=0.5;m=0.5;

for i=0.1:0.2:1

for j=0.1:0.2:1

for k=0.1:0.2:1

[t,y]=ode45(@(t,y) zijian(t,y,b,Cl,Ct,u,w2,I,T1,T2,A,Up,m),[0 50],[i j k]);

%plot3(y(:,1),y(:,2),y(:,3),'linewidth',1);

plot3(y(:,1),y(:,2),y(:,3),'k','linewidth',1); %

set(gca,'XTick',[0:0.2:1],'YTick',[0:0.2:1],'ZTick',[0:0.2:1])

hold on

axis([0 1 0 1 0 1])

view([45 10])

end

end

end

grid on

hold on

xlabel('x','Rotation',0);

ylabel('y','Rotation',0);

zlabel('z','Rotation',360,'position',[0 0 1.05]);

title('Results of 50 iterations of evolution','FontWeight','bold','position',[1 0 -0.13]);

Figure 2.

b=30;Cl=45;Ct=35;w2=20;I=30;T1=15;T2=10;A=30;Up=5;u=0.5;m=0.5;

[t,y]=ode45(@(t,y) zijian(t,y,b,Cl,Ct,u,w2,I,T1,T2,A,Up,m),[0 1],[0.2 0.5 0.2]);

points=1:1:length(t);

plot(t,y(:,1),'k:.','linewidth',1,'markersize',5,'markerfacecolor','r','markerindices',points);

hold on;

b=30;Cl=45;Ct=35;w2=20;I=30;T1=15;T2=10;A=30;Up=5;u=0.5;m=0.5;

[t,y]=ode45(@(t,y) zijian(t,y,b,Cl,Ct,u,w2,I,T1,T2,A,Up,m),[0 1],[0.2 0.5 0.5]);

points=1:1:length(t);

plot(t,y(:,1),'k-','linewidth',1,'markersize',5,'markerfacecolor','r','markerindices',points);

hold on;

b=30;Cl=45;Ct=35;w2=20;I=30;T1=15;T2=10;A=30;Up=5;u=0.5;m=0.5;

[t,y]=ode45(@(t,y) zijian(t,y,b,Cl,Ct,u,w2,I,T1,T2,A,Up,m),[0 1],[0.2 0.5 0.8]);

points=1:1:length(t);

plot(t,y(:,1),'k--','linewidth',1,'markersize',5,'markerfacecolor','r','markerindices',points);

hold on;

b=30;Cl=45;Ct=35;w2=20;I=30;T1=15;T2=10;A=30;Up=5;u=0.5;m=0.5;

[t,y]=ode45(@(t,y) zijian(t,y,b,Cl,Ct,u,w2,I,T1,T2,A,Up,m),[0 1],[0.5 0.5 0.2]);

points=1:1:length(t);

plot(t,y(:,1),'k:.','linewidth',1,'markersize',5,'markerfacecolor','r','markerindices',points);

hold on;

b=30;Cl=45;Ct=35;w2=20;I=30;T1=15;T2=10;A=30;Up=5;u=0.5;m=0.5;

[t,y]=ode45(@(t,y) zijian(t,y,b,Cl,Ct,u,w2,I,T1,T2,A,Up,m),[0 1],[0.5 0.5 0.5]);

points=1:1:length(t);

plot(t,y(:,1),'k-','linewidth',1,'markersize',5,'markerfacecolor','r','markerindices',points);

hold on;

b=30;Cl=45;Ct=35;w2=20;I=30;T1=15;T2=10;A=30;Up=5;u=0.5;m=0.5;

[t,y]=ode45(@(t,y) zijian(t,y,b,Cl,Ct,u,w2,I,T1,T2,A,Up,m),[0 1],[0.5 0.5 0.8]);

points=1:1:length(t);

plot(t,y(:,1),'k--','linewidth',1,'markersize',5,'markerfacecolor','r','markerindices',points);

hold on;

b=30;Cl=45;Ct=35;w2=20;I=30;T1=15;T2=10;A=30;Up=5;u=0.5;m=0.5;

[t,y]=ode45(@(t,y) zijian(t,y,b,Cl,Ct,u,w2,I,T1,T2,A,Up,m),[0 1],[0.8 0.5 0.2]);

points=1:1:length(t);

plot(t,y(:,1),'k:.','linewidth',1,'markersize',5,'markerfacecolor','r','markerindices',points);

hold on;

b=30;Cl=45;Ct=35;w2=20;I=30;T1=15;T2=10;A=30;Up=5;u=0.5;m=0.5;

[t,y]=ode45(@(t,y) zijian(t,y,b,Cl,Ct,u,w2,I,T1,T2,A,Up,m),[0 1],[0.8 0.5 0.5]);

points=1:1:length(t);

plot(t,y(:,1),'k-','linewidth',1,'markersize',5,'markerfacecolor','r','markerindices',points);

hold on;

b=30;Cl=45;Ct=35;w2=20;I=30;T1=15;T2=10;A=30;Up=5;u=0.5;m=0.5;

[t,y]=ode45(@(t,y) zijian(t,y,b,Cl,Ct,u,w2,I,T1,T2,A,Up,m),[0 1],[0.8 0.5 0.8]);

points=1:1:length(t);

plot(t,y(:,1),'k--','linewidth',1,'markersize',5,'markerfacecolor','r','markerindices',points);

hold on;

set(0,'defaultfigurecolor','w')

grid on

hold on

xlabel('$T$','interpreter','latex','Rotation',0);

ylabel('$X$','interpreter','latex');

set(gca,'XTick',[0:0.2:1],'YTick',[0:0.2:1])

axis([0 1 0 1]);

legend('z=0.2','z=0.5','z=0.8');

Figure 3.

b=30;Cl=45;Ct=35;w2=20;I=30;T1=15;T2=10;A=30;Up=5;u=0.5;m=0.5;

[t,y]=ode45(@(t,y) zijian(t,y,b,Cl,Ct,u,w2,I,T1,T2,A,Up,m),[0 1],[0.2 0.2 0.5]);

points=1:1:length(t);

plot(t,y(:,1),'k:.','linewidth',1,'markersize',5,'markerfacecolor','r','markerindices',points);

hold on;

b=30;Cl=45;Ct=35;w2=20;I=30;T1=15;T2=10;A=30;Up=5;u=0.5;m=0.5;

[t,y]=ode45(@(t,y) zijian(t,y,b,Cl,Ct,u,w2,I,T1,T2,A,Up,m),[0 1],[0.2 0.5 0.5]);

points=1:1:length(t);

plot(t,y(:,1),'k-','linewidth',1,'markersize',5,'markerfacecolor','r','markerindices',points);

hold on;

b=30;Cl=45;Ct=35;w2=20;I=30;T1=15;T2=10;A=30;Up=5;u=0.5;m=0.5;

[t,y]=ode45(@(t,y) zijian(t,y,b,Cl,Ct,u,w2,I,T1,T2,A,Up,m),[0 1],[0.2 0.8 0.5]);

points=1:1:length(t);

plot(t,y(:,1),'k--','linewidth',1,'markersize',5,'markerfacecolor','r','markerindices',points);

hold on;

b=30;Cl=45;Ct=35;w2=20;I=30;T1=15;T2=10;A=30;Up=5;u=0.5;m=0.5;

[t,y]=ode45(@(t,y) zijian(t,y,b,Cl,Ct,u,w2,I,T1,T2,A,Up,m),[0 1],[0.5 0.2 0.5]);

points=1:1:length(t);

plot(t,y(:,1),'k:.','linewidth',1,'markersize',5,'markerfacecolor','r','markerindices',points);

hold on;

b=30;Cl=45;Ct=35;w2=20;I=30;T1=15;T2=10;A=30;Up=5;u=0.5;m=0.5;

[t,y]=ode45(@(t,y) zijian(t,y,b,Cl,Ct,u,w2,I,T1,T2,A,Up,m),[0 1],[0.5 0.5 0.5]);

points=1:1:length(t);

plot(t,y(:,1),'k-','linewidth',1,'markersize',5,'markerfacecolor','r','markerindices',points);

hold on;

b=30;Cl=45;Ct=35;w2=20;I=30;T1=15;T2=10;A=30;Up=5;u=0.5;m=0.5;

[t,y]=ode45(@(t,y) zijian(t,y,b,Cl,Ct,u,w2,I,T1,T2,A,Up,m),[0 1],[0.5 0.8 0.5]);

points=1:1:length(t);

plot(t,y(:,1),'k--','linewidth',1,'markersize',5,'markerfacecolor','r','markerindices',points);

hold on;

b=30;Cl=45;Ct=35;w2=20;I=30;T1=15;T2=10;A=30;Up=5;u=0.5;m=0.5;

[t,y]=ode45(@(t,y) zijian(t,y,b,Cl,Ct,u,w2,I,T1,T2,A,Up,m),[0 1],[0.8 0.2 0.5]);

points=1:1:length(t);

plot(t,y(:,1),'k:.','linewidth',1,'markersize',5,'markerfacecolor','r','markerindices',points);

hold on;

b=30;Cl=45;Ct=35;w2=20;I=30;T1=15;T2=10;A=30;Up=5;u=0.5;m=0.5;

[t,y]=ode45(@(t,y) zijian(t,y,b,Cl,Ct,u,w2,I,T1,T2,A,Up,m),[0 1],[0.8 0.5 0.5]);

points=1:1:length(t);

plot(t,y(:,1),'k-','linewidth',1,'markersize',5,'markerfacecolor','r','markerindices',points);

hold on;

b=30;Cl=45;Ct=35;w2=20;I=30;T1=15;T2=10;A=30;Up=5;u=0.5;m=0.5;

[t,y]=ode45(@(t,y) zijian(t,y,b,Cl,Ct,u,w2,I,T1,T2,A,Up,m),[0 1],[0.8 0.8 0.5]);

points=1:1:length(t);

plot(t,y(:,1),'k--','linewidth',1,'markersize',5,'markerfacecolor','r','markerindices',points);

hold on;

set(0,'defaultfigurecolor','w')

grid on

hold on

xlabel('$T$','interpreter','latex','Rotation',0);

ylabel('$X$','interpreter','latex');

set(gca,'XTick',[0:0.2:1],'YTick',[0:0.2:1])

axis([0 1 0 1]);

legend('y=0.2','y=0.5','y=0.8');

Figure 4.

b=30;Cl=45;Ct=35;w2=20;I=30;T1=15;T2=10;A=30;Up=5;u=0.5;m=0.5;

[t,y]=ode45(@(t,y) zijian(t,y,b,Cl,Ct,u,w2,I,T1,T2,A,Up,m),[0 1],[0.2 0.2 0.5]);

points=1:1:length(t);

plot(t,y(:,2),'k:.','linewidth',1,'markersize',5,'markerfacecolor','r','markerindices',points);

hold on;

b=30;Cl=45;Ct=35;w2=20;I=30;T1=15;T2=10;A=30;Up=5;u=0.5;m=0.5;

[t,y]=ode45(@(t,y) zijian(t,y,b,Cl,Ct,u,w2,I,T1,T2,A,Up,m),[0 1],[0.5 0.2 0.5]);

points=1:1:length(t);

plot(t,y(:,2),'k-','linewidth',1,'markersize',5,'markerfacecolor','r','markerindices',points);

hold on;

b=30;Cl=45;Ct=35;w2=20;I=30;T1=15;T2=10;A=30;Up=5;u=0.5;m=0.5;

[t,y]=ode45(@(t,y) zijian(t,y,b,Cl,Ct,u,w2,I,T1,T2,A,Up,m),[0 1],[0.8 0.2 0.5]);

points=1:1:length(t);

plot(t,y(:,2),'k--','linewidth',1,'markersize',5,'markerfacecolor','r','markerindices',points);

hold on;

b=30;Cl=45;Ct=35;w2=20;I=30;T1=15;T2=10;A=30;Up=5;u=0.5;m=0.5;

[t,y]=ode45(@(t,y) zijian(t,y,b,Cl,Ct,u,w2,I,T1,T2,A,Up,m),[0 1],[0.2 0.5 0.5]);

points=1:1:length(t);

plot(t,y(:,2),'k:.','linewidth',1,'markersize',5,'markerfacecolor','r','markerindices',points);

hold on;

b=30;Cl=45;Ct=35;w2=20;I=30;T1=15;T2=10;A=30;Up=5;u=0.5;m=0.5;

[t,y]=ode45(@(t,y) zijian(t,y,b,Cl,Ct,u,w2,I,T1,T2,A,Up,m),[0 1],[0.5 0.5 0.5]);

points=1:1:length(t);

plot(t,y(:,2),'k-','linewidth',1,'markersize',5,'markerfacecolor','r','markerindices',points);

hold on;

b=30;Cl=45;Ct=35;w2=20;I=30;T1=15;T2=10;A=30;Up=5;u=0.5;m=0.5;

[t,y]=ode45(@(t,y) zijian(t,y,b,Cl,Ct,u,w2,I,T1,T2,A,Up,m),[0 1],[0.8 0.5 0.5]);

points=1:1:length(t);

plot(t,y(:,2),'k--','linewidth',1,'markersize',5,'markerfacecolor','r','markerindices',points);

hold on;

b=30;Cl=45;Ct=35;w2=20;I=30;T1=15;T2=10;A=30;Up=5;u=0.5;m=0.5;

[t,y]=ode45(@(t,y) zijian(t,y,b,Cl,Ct,u,w2,I,T1,T2,A,Up,m),[0 1],[0.2 0.8 0.5]);

points=1:1:length(t);

plot(t,y(:,2),'k:.','linewidth',1,'markersize',5,'markerfacecolor','r','markerindices',points);

hold on;

b=30;Cl=45;Ct=35;w2=20;I=30;T1=15;T2=10;A=30;Up=5;u=0.5;m=0.5;

[t,y]=ode45(@(t,y) zijian(t,y,b,Cl,Ct,u,w2,I,T1,T2,A,Up,m),[0 1],[0.5 0.8 0.5]);

points=1:1:length(t);

plot(t,y(:,2),'k-','linewidth',1,'markersize',5,'markerfacecolor','r','markerindices',points);

hold on;

b=30;Cl=45;Ct=35;w2=20;I=30;T1=15;T2=10;A=30;Up=5;u=0.5;m=0.5;

[t,y]=ode45(@(t,y) zijian(t,y,b,Cl,Ct,u,w2,I,T1,T2,A,Up,m),[0 1],[0.8 0.8 0.5]);

points=1:1:length(t);

plot(t,y(:,2),'k--','linewidth',1,'markersize',5,'markerfacecolor','r','markerindices',points);

hold on;

set(0,'defaultfigurecolor','w')

grid on

hold on

xlabel('$T$','interpreter','latex','Rotation',0);

ylabel('$Y$','interpreter','latex');

set(gca,'XTick',[0:0.2:1],'YTick',[0:0.2:1])

axis([0 1 0 1]);

legend('x=0.2','x=0.5','x=0.8');

Figure 5.

b=30;Cl=45;Ct=35;w2=20;I=30;T1=15;T2=10;A=30;Up=5;u=0.5;m=0.5;

[t,y]=ode45(@(t,y) zijian(t,y,b,Cl,Ct,u,w2,I,T1,T2,A,Up,m),[0 1],[0.5 0.2 0.2]);

points=1:1:length(t);

plot(t,y(:,2),'k:.','linewidth',1,'markersize',5,'markerfacecolor','r','markerindices',points);

hold on;

b=30;Cl=45;Ct=35;w2=20;I=30;T1=15;T2=10;A=30;Up=5;u=0.5;m=0.5;

[t,y]=ode45(@(t,y) zijian(t,y,b,Cl,Ct,u,w2,I,T1,T2,A,Up,m),[0 1],[0.5 0.2 0.5]);

points=1:1:length(t);

plot(t,y(:,2),'k-','linewidth',1,'markersize',5,'markerfacecolor','r','markerindices',points);

hold on;

b=30;Cl=45;Ct=35;w2=20;I=30;T1=15;T2=10;A=30;Up=5;u=0.5;m=0.5;

[t,y]=ode45(@(t,y) zijian(t,y,b,Cl,Ct,u,w2,I,T1,T2,A,Up,m),[0 1],[0.5 0.2 0.8]);

points=1:1:length(t);

plot(t,y(:,2),'k--','linewidth',1,'markersize',5,'markerfacecolor','r','markerindices',points);

hold on;

b=30;Cl=45;Ct=35;w2=20;I=30;T1=15;T2=10;A=30;Up=5;u=0.5;m=0.5;

[t,y]=ode45(@(t,y) zijian(t,y,b,Cl,Ct,u,w2,I,T1,T2,A,Up,m),[0 1],[0.5 0.5 0.2]);

points=1:1:length(t);

plot(t,y(:,2),'k:.','linewidth',1,'markersize',5,'markerfacecolor','r','markerindices',points);

hold on;

b=30;Cl=45;Ct=35;w2=20;I=30;T1=15;T2=10;A=30;Up=5;u=0.5;m=0.5;

[t,y]=ode45(@(t,y) zijian(t,y,b,Cl,Ct,u,w2,I,T1,T2,A,Up,m),[0 1],[0.5 0.5 0.5]);

points=1:1:length(t);

plot(t,y(:,2),'k-','linewidth',1,'markersize',5,'markerfacecolor','r','markerindices',points);

hold on;

b=30;Cl=45;Ct=35;w2=20;I=30;T1=15;T2=10;A=30;Up=5;u=0.5;m=0.5;

[t,y]=ode45(@(t,y) zijian(t,y,b,Cl,Ct,u,w2,I,T1,T2,A,Up,m),[0 1],[0.5 0.5 0.8]);

points=1:1:length(t);

plot(t,y(:,2),'k--','linewidth',1,'markersize',5,'markerfacecolor','r','markerindices',points);

hold on;

b=30;Cl=45;Ct=35;w2=20;I=30;T1=15;T2=10;A=30;Up=5;u=0.5;m=0.5;

[t,y]=ode45(@(t,y) zijian(t,y,b,Cl,Ct,u,w2,I,T1,T2,A,Up,m),[0 1],[0.5 0.8 0.2]);

points=1:1:length(t);

plot(t,y(:,2),'k:.','linewidth',1,'markersize',5,'markerfacecolor','r','markerindices',points);

hold on;

b=30;Cl=45;Ct=35;w2=20;I=30;T1=15;T2=10;A=30;Up=5;u=0.5;m=0.5;

[t,y]=ode45(@(t,y) zijian(t,y,b,Cl,Ct,u,w2,I,T1,T2,A,Up,m),[0 1],[0.5 0.8 0.5]);

points=1:1:length(t);

plot(t,y(:,2),'k-','linewidth',1,'markersize',5,'markerfacecolor','r','markerindices',points);

hold on;

b=30;Cl=45;Ct=35;w2=20;I=30;T1=15;T2=10;A=30;Up=5;u=0.5;m=0.5;

[t,y]=ode45(@(t,y) zijian(t,y,b,Cl,Ct,u,w2,I,T1,T2,A,Up,m),[0 1],[0.5 0.8 0.8]);

points=1:1:length(t);

plot(t,y(:,2),'k--','linewidth',1,'markersize',5,'markerfacecolor','r','markerindices',points);

hold on;

set(0,'defaultfigurecolor','w')

grid on

hold on

xlabel('$T$','interpreter','latex','Rotation',0);

ylabel('$Y$','interpreter','latex');

set(gca,'XTick',[0:0.2:1],'YTick',[0:0.2:1])

axis([0 1 0 1]);

legend('z=0.2','z=0.5','z=0.8');

Figure 6.

b=30;Cl=45;Ct=35;w2=20;I=30;T1=15;T2=10;A=30;Up=5;u=0.5;m=0.5;

[t,y]=ode45(@(t,y) zijian(t,y,b,Cl,Ct,u,w2,I,T1,T2,A,Up,m),[0 1],[0.2 0.5 0.2]);

points=1:1:length(t);

plot(t,y(:,3),'k:.','linewidth',1,'markersize',5,'markerfacecolor','r','markerindices',points);

hold on;

b=30;Cl=45;Ct=35;w2=20;I=30;T1=15;T2=10;A=30;Up=5;u=0.5;m=0.5;

[t,y]=ode45(@(t,y) zijian(t,y,b,Cl,Ct,u,w2,I,T1,T2,A,Up,m),[0 1],[0.5 0.5 0.2]);

points=1:1:length(t);

plot(t,y(:,3),'k-','linewidth',1,'markersize',5,'markerfacecolor','r','markerindices',points);

hold on;

b=30;Cl=45;Ct=35;w2=20;I=30;T1=15;T2=10;A=30;Up=5;u=0.5;m=0.5;

[t,y]=ode45(@(t,y) zijian(t,y,b,Cl,Ct,u,w2,I,T1,T2,A,Up,m),[0 1],[0.8 0.5 0.2]);

points=1:1:length(t);

plot(t,y(:,3),'k--','linewidth',1,'markersize',5,'markerfacecolor','r','markerindices',points);

hold on;

b=30;Cl=45;Ct=35;w2=20;I=30;T1=15;T2=10;A=30;Up=5;u=0.5;m=0.5;

[t,y]=ode45(@(t,y) zijian(t,y,b,Cl,Ct,u,w2,I,T1,T2,A,Up,m),[0 1],[0.2 0.5 0.5]);

points=1:1:length(t);

plot(t,y(:,3),'k:.','linewidth',1,'markersize',5,'markerfacecolor','r','markerindices',points);

hold on;

b=30;Cl=45;Ct=35;w2=20;I=30;T1=15;T2=10;A=30;Up=5;u=0.5;m=0.5;

[t,y]=ode45(@(t,y) zijian(t,y,b,Cl,Ct,u,w2,I,T1,T2,A,Up,m),[0 1],[0.5 0.5 0.5]);

points=1:1:length(t);

plot(t,y(:,3),'k-','linewidth',1,'markersize',5,'markerfacecolor','r','markerindices',points);

hold on;

b=30;Cl=45;Ct=35;w2=20;I=30;T1=15;T2=10;A=30;Up=5;u=0.5;m=0.5;

[t,y]=ode45(@(t,y) zijian(t,y,b,Cl,Ct,u,w2,I,T1,T2,A,Up,m),[0 1],[0.8 0.5 0.5]);

points=1:1:length(t);

plot(t,y(:,3),'k--','linewidth',1,'markersize',5,'markerfacecolor','r','markerindices',points);

hold on;

b=30;Cl=45;Ct=35;w2=20;I=30;T1=15;T2=10;A=30;Up=5;u=0.5;m=0.5;

[t,y]=ode45(@(t,y) zijian(t,y,b,Cl,Ct,u,w2,I,T1,T2,A,Up,m),[0 1],[0.2 0.5 0.8]);

points=1:1:length(t);

plot(t,y(:,3),'k:.','linewidth',1,'markersize',5,'markerfacecolor','r','markerindices',points);

hold on;

b=30;Cl=45;Ct=35;w2=20;I=30;T1=15;T2=10;A=30;Up=5;u=0.5;m=0.5;

[t,y]=ode45(@(t,y) zijian(t,y,b,Cl,Ct,u,w2,I,T1,T2,A,Up,m),[0 1],[0.5 0.5 0.8]);

points=1:1:length(t);

plot(t,y(:,3),'k-','linewidth',1,'markersize',5,'markerfacecolor','r','markerindices',points);

hold on;

b=30;Cl=45;Ct=35;w2=20;I=30;T1=15;T2=10;A=30;Up=5;u=0.5;m=0.5;

[t,y]=ode45(@(t,y) zijian(t,y,b,Cl,Ct,u,w2,I,T1,T2,A,Up,m),[0 1],[0.8 0.5 0.8]);

points=1:1:length(t);

plot(t,y(:,3),'k--','linewidth',1,'markersize',5,'markerfacecolor','r','markerindices',points);

hold on;

set(0,'defaultfigurecolor','w')

grid on

hold on

xlabel('$T$','interpreter','latex','Rotation',0);

ylabel('$Z$','interpreter','latex');

set(gca,'XTick',[0:0.2:1],'YTick',[0:0.2:1])

axis([0 1 0 1]);

legend('x=0.2','x=0.5','x=0.8');

Figure 7.

b=30;Cl=45;Ct=35;w2=20;I=30;T1=15;T2=10;A=30;Up=5;u=0.5;m=0.5;

[t,y]=ode45(@(t,y) zijian(t,y,b,Cl,Ct,u,w2,I,T1,T2,A,Up,m),[0 1],[0.5 0.2 0.2]);

points=1:1:length(t);

plot(t,y(:,3),'k:.','linewidth',1,'markersize',5,'markerfacecolor','r','markerindices',points);

hold on;

b=30;Cl=45;Ct=35;w2=20;I=30;T1=15;T2=10;A=30;Up=5;u=0.5;m=0.5;

[t,y]=ode45(@(t,y) zijian(t,y,b,Cl,Ct,u,w2,I,T1,T2,A,Up,m),[0 1],[0.5 0.5 0.2]);

points=1:1:length(t);

plot(t,y(:,3),'k-','linewidth',1,'markersize',5,'markerfacecolor','r','markerindices',points);

hold on;

b=30;Cl=45;Ct=35;w2=20;I=30;T1=15;T2=10;A=30;Up=5;u=0.5;m=0.5;

[t,y]=ode45(@(t,y) zijian(t,y,b,Cl,Ct,u,w2,I,T1,T2,A,Up,m),[0 1],[0.5 0.8 0.2]);

points=1:1:length(t);

plot(t,y(:,3),'k--','linewidth',1,'markersize',5,'markerfacecolor','r','markerindices',points);

hold on;

b=30;Cl=45;Ct=35;w2=20;I=30;T1=15;T2=10;A=30;Up=5;u=0.5;m=0.5;

[t,y]=ode45(@(t,y) zijian(t,y,b,Cl,Ct,u,w2,I,T1,T2,A,Up,m),[0 1],[0.5 0.2 0.5]);

points=1:1:length(t);

plot(t,y(:,3),'k:.','linewidth',1,'markersize',5,'markerfacecolor','r','markerindices',points);

hold on;

b=30;Cl=45;Ct=35;w2=20;I=30;T1=15;T2=10;A=30;Up=5;u=0.5;m=0.5;

[t,y]=ode45(@(t,y) zijian(t,y,b,Cl,Ct,u,w2,I,T1,T2,A,Up,m),[0 1],[0.5 0.5 0.5]);

points=1:1:length(t);

plot(t,y(:,3),'k-','linewidth',1,'markersize',5,'markerfacecolor','r','markerindices',points);

hold on;

b=30;Cl=45;Ct=35;w2=20;I=30;T1=15;T2=10;A=30;Up=5;u=0.5;m=0.5;

[t,y]=ode45(@(t,y) zijian(t,y,b,Cl,Ct,u,w2,I,T1,T2,A,Up,m),[0 1],[0.5 0.8 0.5]);

points=1:1:length(t);

plot(t,y(:,3),'k--','linewidth',1,'markersize',5,'markerfacecolor','r','markerindices',points);

hold on;

b=30;Cl=45;Ct=35;w2=20;I=30;T1=15;T2=10;A=30;Up=5;u=0.5;m=0.5;

[t,y]=ode45(@(t,y) zijian(t,y,b,Cl,Ct,u,w2,I,T1,T2,A,Up,m),[0 1],[0.2 0.2 0.8]);

points=1:1:length(t);

plot(t,y(:,3),'k:.','linewidth',1,'markersize',5,'markerfacecolor','r','markerindices',points);

hold on;

b=30;Cl=45;Ct=35;w2=20;I=30;T1=15;T2=10;A=30;Up=5;u=0.5;m=0.5;

[t,y]=ode45(@(t,y) zijian(t,y,b,Cl,Ct,u,w2,I,T1,T2,A,Up,m),[0 1],[0.5 0.5 0.8]);

points=1:1:length(t);

plot(t,y(:,3),'k-','linewidth',1,'markersize',5,'markerfacecolor','r','markerindices',points);

hold on;

b=30;Cl=45;Ct=35;w2=20;I=30;T1=15;T2=10;A=30;Up=5;u=0.5;m=0.5;

[t,y]=ode45(@(t,y) zijian(t,y,b,Cl,Ct,u,w2,I,T1,T2,A,Up,m),[0 1],[0.8 0.8 0.8]);

points=1:1:length(t);

plot(t,y(:,3),'k--','linewidth',1,'markersize',5,'markerfacecolor','r','markerindices',points);

hold on;

set(0,'defaultfigurecolor','w')

grid on

hold on

xlabel('$T$','interpreter','latex','Rotation',0);

ylabel('$Z$','interpreter','latex');

set(gca,'XTick',[0:0.2:1],'YTick',[0:0.2:1])

axis([0 1 0 1]);

legend('y=0.2','y=0.5','y=0.8');

Figure 8.

b=30;Cl=45;Ct=35;w2=20;I=30;T1=15;T2=10;A=30;Up=5;u=0.2;m=0.5;

[t,y]=ode45(@(t,y) zijian(t,y,b,Cl,Ct,u,w2,I,T1,T2,A,Up,m),[0 1],[0.2 0.5 0.5]);

points=1:1:length(t);

plot(t,y(:,1),'k:.','linewidth',1,'markersize',5,'markerfacecolor','r','markerindices',points);

hold on;

b=30;Cl=45;Ct=35;w2=20;I=30;T1=15;T2=10;A=30;Up=5;u=0.5;m=0.5;

[t,y]=ode45(@(t,y) zijian(t,y,b,Cl,Ct,u,w2,I,T1,T2,A,Up,m),[0 1],[0.2 0.5 0.5]);

points=1:1:length(t);

plot(t,y(:,1),'k-','linewidth',1,'markersize',5,'markerfacecolor','r','markerindices',points);

hold on;

b=30;Cl=45;Ct=35;w2=20;I=30;T1=15;T2=10;A=30;Up=5;u=0.8;m=0.5;

[t,y]=ode45(@(t,y) zijian(t,y,b,Cl,Ct,u,w2,I,T1,T2,A,Up,m),[0 1],[0.2 0.5 0.5]);

points=1:1:length(t);

plot(t,y(:,1),'k--','linewidth',1,'markersize',5,'markerfacecolor','r','markerindices',points);

hold on;

b=30;Cl=45;Ct=35;w2=20;I=30;T1=15;T2=10;A=30;Up=5;u=0.2;m=0.5;

[t,y]=ode45(@(t,y) zijian(t,y,b,Cl,Ct,u,w2,I,T1,T2,A,Up,m),[0 1],[0.5 0.5 0.5]);

points=1:1:length(t);

plot(t,y(:,1),'k:.','linewidth',1,'markersize',5,'markerfacecolor','r','markerindices',points);

hold on;

b=30;Cl=45;Ct=35;w2=20;I=30;T1=15;T2=10;A=30;Up=5;u=0.5;m=0.5;

[t,y]=ode45(@(t,y) zijian(t,y,b,Cl,Ct,u,w2,I,T1,T2,A,Up,m),[0 1],[0.5 0.5 0.5]);

points=1:1:length(t);

plot(t,y(:,1),'k-','linewidth',1,'markersize',5,'markerfacecolor','r','markerindices',points);

hold on;

b=30;Cl=45;Ct=35;w2=20;I=30;T1=15;T2=10;A=30;Up=5;u=0.8;m=0.5;

[t,y]=ode45(@(t,y) zijian(t,y,b,Cl,Ct,u,w2,I,T1,T2,A,Up,m),[0 1],[0.5 0.5 0.5]);

points=1:1:length(t);

plot(t,y(:,1),'k--','linewidth',1,'markersize',5,'markerfacecolor','r','markerindices',points);

hold on;

b=30;Cl=45;Ct=35;w2=20;I=30;T1=15;T2=10;A=30;Up=5;u=0.2;m=0.5;

[t,y]=ode45(@(t,y) zijian(t,y,b,Cl,Ct,u,w2,I,T1,T2,A,Up,m),[0 1],[0.8 0.5 0.5]);

points=1:1:length(t);

plot(t,y(:,1),'k:.','linewidth',1,'markersize',5,'markerfacecolor','r','markerindices',points);

hold on;

b=30;Cl=45;Ct=35;w2=20;I=30;T1=15;T2=10;A=30;Up=5;u=0.5;m=0.5;

[t,y]=ode45(@(t,y) zijian(t,y,b,Cl,Ct,u,w2,I,T1,T2,A,Up,m),[0 1],[0.8 0.5 0.5]);

points=1:1:length(t);

plot(t,y(:,1),'k-','linewidth',1,'markersize',5,'markerfacecolor','r','markerindices',points);

hold on;

b=30;Cl=45;Ct=35;w2=20;I=30;T1=15;T2=10;A=30;Up=5;u=0.8;m=0.5;

[t,y]=ode45(@(t,y) zijian(t,y,b,Cl,Ct,u,w2,I,T1,T2,A,Up,m),[0 1],[0.8 0.5 0.5]);

points=1:1:length(t);

plot(t,y(:,1),'k--','linewidth',1,'markersize',5,'markerfacecolor','r','markerindices',points);

hold on;

set(0,'defaultfigurecolor','w')

grid on

hold on

xlabel('$T$','interpreter','latex','Rotation',0);

ylabel('$X$','interpreter','latex');

set(gca,'XTick',[0:0.2:1],'YTick',[0:0.2:1])

axis([0 1 0 1]);

legend('u=0.2','u=0.5','u=0.8');

Figure 9.

b=30;Cl=45;Ct=35;w2=20;I=30;T1=15;T2=10;A=30;Up=5;u=0.2;m=0.5;

[t,y]=ode45(@(t,y) zijian(t,y,b,Cl,Ct,u,w2,I,T1,T2,A,Up,m),[0 1],[0.5 0.2 0.5]);

points=1:1:length(t);

plot(t,y(:,2),'k:.','linewidth',1,'markersize',5,'markerfacecolor','r','markerindices',points);

hold on;

b=30;Cl=45;Ct=35;w2=20;I=30;T1=15;T2=10;A=30;Up=5;u=0.5;m=0.5;

[t,y]=ode45(@(t,y) zijian(t,y,b,Cl,Ct,u,w2,I,T1,T2,A,Up,m),[0 1],[0.5 0.2 0.5]);

points=1:1:length(t);

plot(t,y(:,2),'k-','linewidth',1,'markersize',5,'markerfacecolor','r','markerindices',points);

hold on;

b=30;Cl=45;Ct=35;w2=20;I=30;T1=15;T2=10;A=30;Up=5;u=0.8;m=0.5;

[t,y]=ode45(@(t,y) zijian(t,y,b,Cl,Ct,u,w2,I,T1,T2,A,Up,m),[0 1],[0.5 0.2 0.5]);

points=1:1:length(t);

plot(t,y(:,2),'k--','linewidth',1,'markersize',5,'markerfacecolor','r','markerindices',points);

hold on;

b=30;Cl=45;Ct=35;w2=20;I=30;T1=15;T2=10;A=30;Up=5;u=0.2;m=0.5;

[t,y]=ode45(@(t,y) zijian(t,y,b,Cl,Ct,u,w2,I,T1,T2,A,Up,m),[0 1],[0.5 0.5 0.5]);

points=1:1:length(t);

plot(t,y(:,2),'k:.','linewidth',1,'markersize',5,'markerfacecolor','r','markerindices',points);

hold on;

b=30;Cl=45;Ct=35;w2=20;I=30;T1=15;T2=10;A=30;Up=5;u=0.5;m=0.5;

[t,y]=ode45(@(t,y) zijian(t,y,b,Cl,Ct,u,w2,I,T1,T2,A,Up,m),[0 1],[0.5 0.5 0.5]);

points=1:1:length(t);

plot(t,y(:,2),'k-','linewidth',1,'markersize',5,'markerfacecolor','r','markerindices',points);

hold on;

b=30;Cl=45;Ct=35;w2=20;I=30;T1=15;T2=10;A=30;Up=5;u=0.8;m=0.5;

[t,y]=ode45(@(t,y) zijian(t,y,b,Cl,Ct,u,w2,I,T1,T2,A,Up,m),[0 1],[0.5 0.5 0.5]);

points=1:1:length(t);

plot(t,y(:,2),'k--','linewidth',1,'markersize',5,'markerfacecolor','r','markerindices',points);

hold on;

b=30;Cl=45;Ct=35;w2=20;I=30;T1=15;T2=10;A=30;Up=5;u=0.2;m=0.5;

[t,y]=ode45(@(t,y) zijian(t,y,b,Cl,Ct,u,w2,I,T1,T2,A,Up,m),[0 1],[0.5 0.8 0.5]);

points=1:1:length(t);

plot(t,y(:,2),'k:.','linewidth',1,'markersize',5,'markerfacecolor','r','markerindices',points);

hold on;

b=30;Cl=45;Ct=35;w2=20;I=30;T1=15;T2=10;A=30;Up=5;u=0.5;m=0.5;

[t,y]=ode45(@(t,y) zijian(t,y,b,Cl,Ct,u,w2,I,T1,T2,A,Up,m),[0 1],[0.5 0.8 0.5]);

points=1:1:length(t);

plot(t,y(:,2),'k-','linewidth',1,'markersize',5,'markerfacecolor','r','markerindices',points);

hold on;

b=30;Cl=45;Ct=35;w2=20;I=30;T1=15;T2=10;A=30;Up=5;u=0.8;m=0.5;

[t,y]=ode45(@(t,y) zijian(t,y,b,Cl,Ct,u,w2,I,T1,T2,A,Up,m),[0 1],[0.5 0.8 0.5]);

points=1:1:length(t);

plot(t,y(:,2),'k--','linewidth',1,'markersize',5,'markerfacecolor','r','markerindices',points);

hold on;

set(0,'defaultfigurecolor','w')

grid on

hold on

xlabel('$T$','interpreter','latex','Rotation',0);

ylabel('$Y$','interpreter','latex');

set(gca,'XTick',[0:0.2:1],'YTick',[0:0.2:1])

axis([0 1 0 1]);

legend('u=0.2','u=0.5','u=0.8');

Figure 10.

b=30;Cl=45;Ct=35;w2=20;I=30;T1=15;T2=10;A=30;Up=5;u=0.2;m=0.5;

[t,y]=ode45(@(t,y) zijian(t,y,b,Cl,Ct,u,w2,I,T1,T2,A,Up,m),[0 1],[0.5 0.5 0.2]);

points=1:1:length(t);

plot(t,y(:,3),'k:.','linewidth',1,'markersize',5,'markerfacecolor','r','markerindices',points);

hold on;

b=30;Cl=45;Ct=35;w2=20;I=30;T1=15;T2=10;A=30;Up=5;u=0.5;m=0.5;

[t,y]=ode45(@(t,y) zijian(t,y,b,Cl,Ct,u,w2,I,T1,T2,A,Up,m),[0 1],[0.5 0.5 0.2]);

points=1:1:length(t);

plot(t,y(:,3),'k-','linewidth',1,'markersize',5,'markerfacecolor','r','markerindices',points);

hold on;

b=30;Cl=45;Ct=35;w2=20;I=30;T1=15;T2=10;A=30;Up=5;u=0.8;m=0.5;

[t,y]=ode45(@(t,y) zijian(t,y,b,Cl,Ct,u,w2,I,T1,T2,A,Up,m),[0 1],[0.5 0.5 0.2]);

points=1:1:length(t);

plot(t,y(:,3),'k--','linewidth',1,'markersize',5,'markerfacecolor','r','markerindices',points);

hold on;

b=30;Cl=45;Ct=35;w2=20;I=30;T1=15;T2=10;A=30;Up=5;u=0.2;m=0.5;

[t,y]=ode45(@(t,y) zijian(t,y,b,Cl,Ct,u,w2,I,T1,T2,A,Up,m),[0 1],[0.5 0.5 0.5]);

points=1:1:length(t);

plot(t,y(:,3),'k:.','linewidth',1,'markersize',5,'markerfacecolor','r','markerindices',points);

hold on;

b=30;Cl=45;Ct=35;w2=20;I=30;T1=15;T2=10;A=30;Up=5;u=0.5;m=0.5;

[t,y]=ode45(@(t,y) zijian(t,y,b,Cl,Ct,u,w2,I,T1,T2,A,Up,m),[0 1],[0.5 0.5 0.5]);

points=1:1:length(t);

plot(t,y(:,3),'k-','linewidth',1,'markersize',5,'markerfacecolor','r','markerindices',points);

hold on;

b=30;Cl=45;Ct=35;w2=20;I=30;T1=15;T2=10;A=30;Up=5;u=0.8;m=0.5;

[t,y]=ode45(@(t,y) zijian(t,y,b,Cl,Ct,u,w2,I,T1,T2,A,Up,m),[0 1],[0.5 0.5 0.5]);

points=1:1:length(t);

plot(t,y(:,3),'k--','linewidth',1,'markersize',5,'markerfacecolor','r','markerindices',points);

hold on;

b=30;Cl=45;Ct=35;w2=20;I=30;T1=15;T2=10;A=30;Up=5;u=0.2;m=0.5;

[t,y]=ode45(@(t,y) zijian(t,y,b,Cl,Ct,u,w2,I,T1,T2,A,Up,m),[0 1],[0.5 0.5 0.8]);

points=1:1:length(t);

plot(t,y(:,3),'k:.','linewidth',1,'markersize',5,'markerfacecolor','r','markerindices',points);

hold on;

b=30;Cl=45;Ct=35;w2=20;I=30;T1=15;T2=10;A=30;Up=5;u=0.5;m=0.5;

[t,y]=ode45(@(t,y) zijian(t,y,b,Cl,Ct,u,w2,I,T1,T2,A,Up,m),[0 1],[0.5 0.5 0.8]);

points=1:1:length(t);

plot(t,y(:,3),'k-','linewidth',1,'markersize',5,'markerfacecolor','r','markerindices',points);

hold on;

b=30;Cl=45;Ct=35;w2=20;I=30;T1=15;T2=10;A=30;Up=5;u=0.8;m=0.5;

[t,y]=ode45(@(t,y) zijian(t,y,b,Cl,Ct,u,w2,I,T1,T2,A,Up,m),[0 1],[0.5 0.5 0.8]);

points=1:1:length(t);

plot(t,y(:,3),'k--','linewidth',1,'markersize',5,'markerfacecolor','r','markerindices',points);

hold on;

set(0,'defaultfigurecolor','w')

grid on

hold on

xlabel('$T$','interpreter','latex','Rotation',0);

ylabel('$Z$','interpreter','latex');

set(gca,'XTick',[0:0.2:1],'YTick',[0:0.2:1])

axis([0 1 0 1]);

legend('u=0.2','u=0.5','u=0.8');

Figure 11.

b=30;Cl=45;Ct=35;w2=20;I=30;T1=15;T2=10;A=30;Up=5;u=0.5;m=0.8;

[t,y]=ode45(@(t,y) zijian(t,y,b,Cl,Ct,u,w2,I,T1,T2,A,Up,m),[0 1],[0.2 0.5 0.5]);

points=1:1:length(t);

plot(t,y(:,1),'k:.','linewidth',1,'markersize',5,'markerfacecolor','r','markerindices',points);

hold on;

b=30;Cl=45;Ct=35;w2=20;I=30;T1=15;T2=10;A=30;Up=5;u=0.5;m=0.5;

[t,y]=ode45(@(t,y) zijian(t,y,b,Cl,Ct,u,w2,I,T1,T2,A,Up,m),[0 1],[0.2 0.5 0.5]);

points=1:1:length(t);

plot(t,y(:,1),'k-','linewidth',1,'markersize',5,'markerfacecolor','r','markerindices',points);

hold on;

b=30;Cl=45;Ct=35;w2=20;I=30;T1=15;T2=10;A=30;Up=5;u=0.5;m=0.2;

[t,y]=ode45(@(t,y) zijian(t,y,b,Cl,Ct,u,w2,I,T1,T2,A,Up,m),[0 1],[0.2 0.5 0.5]);

points=1:1:length(t);

plot(t,y(:,1),'k--','linewidth',1,'markersize',5,'markerfacecolor','r','markerindices',points);

hold on;

b=30;Cl=45;Ct=35;w2=20;I=30;T1=15;T2=10;A=30;Up=5;u=0.5;m=0.8;

[t,y]=ode45(@(t,y) zijian(t,y,b,Cl,Ct,u,w2,I,T1,T2,A,Up,m),[0 1],[0.5 0.5 0.5]);

points=1:1:length(t);

plot(t,y(:,1),'k:.','linewidth',1,'markersize',5,'markerfacecolor','r','markerindices',points);

hold on;

b=30;Cl=45;Ct=35;w2=20;I=30;T1=15;T2=10;A=30;Up=5;u=0.5;m=0.5;

[t,y]=ode45(@(t,y) zijian(t,y,b,Cl,Ct,u,w2,I,T1,T2,A,Up,m),[0 1],[0.5 0.5 0.5]);

points=1:1:length(t);

plot(t,y(:,1),'k-','linewidth',1,'markersize',5,'markerfacecolor','r','markerindices',points);

hold on;

b=30;Cl=45;Ct=35;w2=20;I=30;T1=15;T2=10;A=30;Up=5;u=0.5;m=0.2;

[t,y]=ode45(@(t,y) zijian(t,y,b,Cl,Ct,u,w2,I,T1,T2,A,Up,m),[0 1],[0.5 0.5 0.5]);

points=1:1:length(t);

plot(t,y(:,1),'k--','linewidth',1,'markersize',5,'markerfacecolor','r','markerindices',points);

hold on;

b=30;Cl=45;Ct=35;w2=20;I=30;T1=15;T2=10;A=30;Up=5;u=0.5;m=0.8;

[t,y]=ode45(@(t,y) zijian(t,y,b,Cl,Ct,u,w2,I,T1,T2,A,Up,m),[0 1],[0.8 0.5 0.5]);

points=1:1:length(t);

plot(t,y(:,3),'k:.','linewidth',1,'markersize',5,'markerfacecolor','r','markerindices',points);

hold on;

b=30;Cl=45;Ct=35;w2=20;I=30;T1=15;T2=10;A=30;Up=5;u=0.5;m=0.5;

[t,y]=ode45(@(t,y) zijian(t,y,b,Cl,Ct,u,w2,I,T1,T2,A,Up,m),[0 1],[0.8 0.5 0.5]);

points=1:1:length(t);

plot(t,y(:,1),'k-','linewidth',1,'markersize',5,'markerfacecolor','r','markerindices',points);

hold on;

b=30;Cl=45;Ct=35;w2=20;I=30;T1=15;T2=10;A=30;Up=5;u=0.5;m=0.2;

[t,y]=ode45(@(t,y) zijian(t,y,b,Cl,Ct,u,w2,I,T1,T2,A,Up,m),[0 1],[0.8 0.5 0.5]);

points=1:1:length(t);

plot(t,y(:,1),'k--','linewidth',1,'markersize',5,'markerfacecolor','r','markerindices',points);

hold on;

set(0,'defaultfigurecolor','w')

grid on

hold on

xlabel('$T$','interpreter','latex','Rotation',0);

ylabel('$X$','interpreter','latex');

set(gca,'XTick',[0:0.2:1],'YTick',[0:0.2:1])

axis([0 1 0 1]);

legend('1-m=0.2','1-m=0.5','1-m=0.8');

Figure 12.

b=30;Cl=45;Ct=35;w2=20;I=30;T1=15;T2=10;A=30;Up=5;u=0.5;m=0.8;

[t,y]=ode45(@(t,y) zijian(t,y,b,Cl,Ct,u,w2,I,T1,T2,A,Up,m),[0 1],[0.5 0.5 0.2]);

points=1:1:length(t);

plot(t,y(:,3),'k:.','linewidth',1,'markersize',5,'markerfacecolor','r','markerindices',points);

hold on;

b=30;Cl=45;Ct=35;w2=20;I=30;T1=15;T2=10;A=30;Up=5;u=0.5;m=0.5;

[t,y]=ode45(@(t,y) zijian(t,y,b,Cl,Ct,u,w2,I,T1,T2,A,Up,m),[0 1],[0.5 0.5 0.2]);

points=1:1:length(t);

plot(t,y(:,3),'k-','linewidth',1,'markersize',5,'markerfacecolor','r','markerindices',points);

hold on;

b=30;Cl=45;Ct=35;w2=20;I=30;T1=15;T2=10;A=30;Up=5;u=0.5;m=0.2;

[t,y]=ode45(@(t,y) zijian(t,y,b,Cl,Ct,u,w2,I,T1,T2,A,Up,m),[0 1],[0.5 0.5 0.2]);

points=1:1:length(t);

plot(t,y(:,3),'k--','linewidth',1,'markersize',5,'markerfacecolor','r','markerindices',points);

hold on;

b=30;Cl=45;Ct=35;w2=20;I=30;T1=15;T2=10;A=30;Up=5;u=0.5;m=0.8;

[t,y]=ode45(@(t,y) zijian(t,y,b,Cl,Ct,u,w2,I,T1,T2,A,Up,m),[0 1],[0.5 0.5 0.5]);

points=1:1:length(t);

plot(t,y(:,3),'k:.','linewidth',1,'markersize',5,'markerfacecolor','r','markerindices',points);

hold on;

b=30;Cl=45;Ct=35;w2=20;I=30;T1=15;T2=10;A=30;Up=5;u=0.5;m=0.5;

[t,y]=ode45(@(t,y) zijian(t,y,b,Cl,Ct,u,w2,I,T1,T2,A,Up,m),[0 1],[0.5 0.5 0.5]);

points=1:1:length(t);

plot(t,y(:,3),'k-','linewidth',1,'markersize',5,'markerfacecolor','r','markerindices',points);

hold on;

b=30;Cl=45;Ct=35;w2=20;I=30;T1=15;T2=10;A=30;Up=5;u=0.5;m=0.2;

[t,y]=ode45(@(t,y) zijian(t,y,b,Cl,Ct,u,w2,I,T1,T2,A,Up,m),[0 1],[0.5 0.5 0.5]);

points=1:1:length(t);

plot(t,y(:,3),'k--','linewidth',1,'markersize',5,'markerfacecolor','r','markerindices',points);

hold on;

b=30;Cl=45;Ct=35;w2=20;I=30;T1=15;T2=10;A=30;Up=5;u=0.5;m=0.8;

[t,y]=ode45(@(t,y) zijian(t,y,b,Cl,Ct,u,w2,I,T1,T2,A,Up,m),[0 1],[0.5 0.5 0.8]);

points=1:1:length(t);

plot(t,y(:,3),'k:.','linewidth',1,'markersize',5,'markerfacecolor','r','markerindices',points);

hold on;

b=30;Cl=45;Ct=35;w2=20;I=30;T1=15;T2=10;A=30;Up=5;u=0.5;m=0.5;

[t,y]=ode45(@(t,y) zijian(t,y,b,Cl,Ct,u,w2,I,T1,T2,A,Up,m),[0 1],[0.5 0.5 0.8]);

points=1:1:length(t);

plot(t,y(:,3),'k-','linewidth',1,'markersize',5,'markerfacecolor','r','markerindices',points);

hold on;

b=30;Cl=45;Ct=35;w2=20;I=30;T1=15;T2=10;A=30;Up=5;u=0.5;m=0.2;

[t,y]=ode45(@(t,y) zijian(t,y,b,Cl,Ct,u,w2,I,T1,T2,A,Up,m),[0 1],[0.5 0.5 0.8]);

points=1:1:length(t);

plot(t,y(:,3),'k--','linewidth',1,'markersize',5,'markerfacecolor','r','markerindices',points);

hold on;

set(0,'defaultfigurecolor','w')

grid on

hold on

xlabel('$T$','interpreter','latex','Rotation',0);

ylabel('$Z$','interpreter','latex');

set(gca,'XTick',[0:0.2:1],'YTick',[0:0.2:1])

axis([0 1 0 1]);

legend('1-m=0.2','1-m=0.5','1-m=0.8');

Figure 13.

b=30;Cl=45;Ct=35;w2=20;I=30;T1=15;T2=10;A=30;Up=5;u=0.5;m=0.8;

[t,y]=ode45(@(t,y) zijian(t,y,b,Cl,Ct,u,w2,I,T1,T2,A,Up,m),[0 1],[0.5 0.5 0.2]);

points=1:1:length(t);

plot(t,y(:,3),'k:.','linewidth',1,'markersize',5,'markerfacecolor','r','markerindices',points);

hold on;

b=30;Cl=45;Ct=35;w2=20;I=30;T1=15;T2=10;A=30;Up=5;u=0.5;m=0.5;

[t,y]=ode45(@(t,y) zijian(t,y,b,Cl,Ct,u,w2,I,T1,T2,A,Up,m),[0 1],[0.5 0.5 0.2]);

points=1:1:length(t);

plot(t,y(:,3),'k-','linewidth',1,'markersize',5,'markerfacecolor','r','markerindices',points);

hold on;

b=30;Cl=45;Ct=35;w2=20;I=30;T1=15;T2=10;A=30;Up=5;u=0.5;m=0.2;

[t,y]=ode45(@(t,y) zijian(t,y,b,Cl,Ct,u,w2,I,T1,T2,A,Up,m),[0 1],[0.5 0.5 0.2]);

points=1:1:length(t);

plot(t,y(:,3),'k--','linewidth',1,'markersize',5,'markerfacecolor','r','markerindices',points);

hold on;

b=30;Cl=45;Ct=35;w2=20;I=30;T1=15;T2=10;A=30;Up=5;u=0.5;m=0.8;

[t,y]=ode45(@(t,y) zijian(t,y,b,Cl,Ct,u,w2,I,T1,T2,A,Up,m),[0 1],[0.5 0.5 0.5]);

points=1:1:length(t);

plot(t,y(:,3),'k:.','linewidth',1,'markersize',5,'markerfacecolor','r','markerindices',points);

hold on;

b=30;Cl=45;Ct=35;w2=20;I=30;T1=15;T2=10;A=30;Up=5;u=0.5;m=0.5;

[t,y]=ode45(@(t,y) zijian(t,y,b,Cl,Ct,u,w2,I,T1,T2,A,Up,m),[0 1],[0.5 0.5 0.5]);

points=1:1:length(t);

plot(t,y(:,3),'k-','linewidth',1,'markersize',5,'markerfacecolor','r','markerindices',points);

hold on;

b=30;Cl=45;Ct=35;w2=20;I=30;T1=15;T2=10;A=30;Up=5;u=0.5;m=0.2;

[t,y]=ode45(@(t,y) zijian(t,y,b,Cl,Ct,u,w2,I,T1,T2,A,Up,m),[0 1],[0.5 0.5 0.5]);

points=1:1:length(t);

plot(t,y(:,3),'k--','linewidth',1,'markersize',5,'markerfacecolor','r','markerindices',points);

hold on;

b=30;Cl=45;Ct=35;w2=20;I=30;T1=15;T2=10;A=30;Up=5;u=0.5;m=0.8;

[t,y]=ode45(@(t,y) zijian(t,y,b,Cl,Ct,u,w2,I,T1,T2,A,Up,m),[0 1],[0.5 0.5 0.8]);

points=1:1:length(t);

plot(t,y(:,3),'k:.','linewidth',1,'markersize',5,'markerfacecolor','r','markerindices',points);

hold on;

b=30;Cl=45;Ct=35;w2=20;I=30;T1=15;T2=10;A=30;Up=5;u=0.5;m=0.5;

[t,y]=ode45(@(t,y) zijian(t,y,b,Cl,Ct,u,w2,I,T1,T2,A,Up,m),[0 1],[0.5 0.5 0.8]);

points=1:1:length(t);

plot(t,y(:,3),'k-','linewidth',1,'markersize',5,'markerfacecolor','r','markerindices',points);

hold on;

b=30;Cl=45;Ct=35;w2=20;I=30;T1=15;T2=10;A=30;Up=5;u=0.5;m=0.2;

[t,y]=ode45(@(t,y) zijian(t,y,b,Cl,Ct,u,w2,I,T1,T2,A,Up,m),[0 1],[0.5 0.5 0.8]);

points=1:1:length(t);

plot(t,y(:,3),'k--','linewidth',1,'markersize',5,'markerfacecolor','r','markerindices',points);

hold on;

set(0,'defaultfigurecolor','w')

grid on

hold on

xlabel('$T$','interpreter','latex','Rotation',0);

ylabel('$Z$','interpreter','latex');

set(gca,'XTick',[0:0.2:1],'YTick',[0:0.2:1])

axis([0 1 0 1]);

legend('1-m=0.2','1-m=0.5','1-m=0.8');

Figure 14.

b=30;Cl=45;Ct=35;w2=20;I=30;T1=15;T2=10;A=30;Up=5;u=0.5;m=0.5;

[t,y]=ode45(@(t,y) zijian(t,y,b,Cl,Ct,u,w2,I,T1,T2,A,Up,m),[0 1],[0.2 0.5 0.5]);

points=1:1:length(t);

plot(t,y(:,1),'k:.','linewidth',1,'markersize',5,'markerfacecolor','r','markerindices',points);

hold on;

b=30;Cl=45;Ct=35;w2=20;I=30;T1=15;T2=10;A=30;Up=10;u=0.5;m=0.5;

[t,y]=ode45(@(t,y) zijian(t,y,b,Cl,Ct,u,w2,I,T1,T2,A,Up,m),[0 1],[0.2 0.5 0.5]);

points=1:1:length(t);

plot(t,y(:,1),'k-','linewidth',1,'markersize',5,'markerfacecolor','r','markerindices',points);

hold on;

b=30;Cl=45;Ct=35;w2=20;I=30;T1=15;T2=10;A=30;Up=15;u=0.5;m=0.5;

[t,y]=ode45(@(t,y) zijian(t,y,b,Cl,Ct,u,w2,I,T1,T2,A,Up,m),[0 1],[0.2 0.5 0.5]);

points=1:1:length(t);

plot(t,y(:,1),'k--','linewidth',1,'markersize',5,'markerfacecolor','r','markerindices',points);

hold on;

b=30;Cl=45;Ct=35;w2=20;I=30;T1=15;T2=10;A=30;Up=5;u=0.5;m=0.5;

[t,y]=ode45(@(t,y) zijian(t,y,b,Cl,Ct,u,w2,I,T1,T2,A,Up,m),[0 1],[0.5 0.5 0.5]);

points=1:1:length(t);

plot(t,y(:,1),'k:.','linewidth',1,'markersize',5,'markerfacecolor','r','markerindices',points);

hold on;

b=30;Cl=45;Ct=35;w2=20;I=30;T1=15;T2=10;A=30;Up=10;u=0.5;m=0.5;

[t,y]=ode45(@(t,y) zijian(t,y,b,Cl,Ct,u,w2,I,T1,T2,A,Up,m),[0 1],[0.5 0.5 0.5]);

points=1:1:length(t);

plot(t,y(:,1),'k-','linewidth',1,'markersize',5,'markerfacecolor','r','markerindices',points);

hold on;

b=30;Cl=45;Ct=35;w2=20;I=30;T1=15;T2=10;A=30;Up=15;u=0.5;m=0.5;

[t,y]=ode45(@(t,y) zijian(t,y,b,Cl,Ct,u,w2,I,T1,T2,A,Up,m),[0 1],[0.5 0.5 0.5]);

points=1:1:length(t);

plot(t,y(:,1),'k--','linewidth',1,'markersize',5,'markerfacecolor','r','markerindices',points);

hold on;

b=30;Cl=45;Ct=35;w2=20;I=30;T1=15;T2=10;A=30;Up=5;u=0.5;m=0.5;

[t,y]=ode45(@(t,y) zijian(t,y,b,Cl,Ct,u,w2,I,T1,T2,A,Up,m),[0 1],[0.8 0.5 0.5]);

points=1:1:length(t);

plot(t,y(:,1),'k:.','linewidth',1,'markersize',5,'markerfacecolor','r','markerindices',points);

hold on;

b=30;Cl=45;Ct=35;w2=20;I=30;T1=15;T2=10;A=30;Up=10;u=0.5;m=0.5;

[t,y]=ode45(@(t,y) zijian(t,y,b,Cl,Ct,u,w2,I,T1,T2,A,Up,m),[0 1],[0.8 0.5 0.5]);

points=1:1:length(t);

plot(t,y(:,1),'k-','linewidth',1,'markersize',5,'markerfacecolor','r','markerindices',points);

hold on;

b=30;Cl=45;Ct=35;w2=20;I=30;T1=15;T2=10;A=30;Up=15;u=0.5;m=0.5;

[t,y]=ode45(@(t,y) zijian(t,y,b,Cl,Ct,u,w2,I,T1,T2,A,Up,m),[0 1],[0.8 0.5 0.5]);

points=1:1:length(t);

plot(t,y(:,1),'k--','linewidth',1,'markersize',5,'markerfacecolor','r','markerindices',points);

hold on;

set(0,'defaultfigurecolor','w')

grid on

hold on

xlabel('$T$','interpreter','latex','Rotation',0);

ylabel('$X$','interpreter','latex');

set(gca,'XTick',[0:0.2:1],'YTick',[0:0.2:1])

axis([0 1 0 1]);

legend('Up=5','Up=10','Up=15');

Figure 15.

b=30;Cl=45;Ct=35;w2=20;I=30;T1=15;T2=10;A=30;Up=5;u=0.5;m=0.5;

[t,y]=ode45(@(t,y) zijian(t,y,b,Cl,Ct,u,w2,I,T1,T2,A,Up,m),[0 1],[0.5 0.5 0.2]);

points=1:1:length(t);

plot(t,y(:,3),'k:.','linewidth',1,'markersize',5,'markerfacecolor','r','markerindices',points);

hold on;

b=30;Cl=45;Ct=35;w2=20;I=30;T1=15;T2=10;A=30;Up=10;u=0.5;m=0.5;

[t,y]=ode45(@(t,y) zijian(t,y,b,Cl,Ct,u,w2,I,T1,T2,A,Up,m),[0 1],[0.5 0.5 0.2]);

points=1:1:length(t);

plot(t,y(:,3),'k-','linewidth',1,'markersize',5,'markerfacecolor','r','markerindices',points);

hold on;

b=30;Cl=45;Ct=35;w2=20;I=30;T1=15;T2=10;A=30;Up=15;u=0.5;m=0.5;

[t,y]=ode45(@(t,y) zijian(t,y,b,Cl,Ct,u,w2,I,T1,T2,A,Up,m),[0 1],[0.5 0.5 0.2]);

points=1:1:length(t);

plot(t,y(:,3),'k--','linewidth',1,'markersize',5,'markerfacecolor','r','markerindices',points);

hold on;

b=30;Cl=45;Ct=35;w2=20;I=30;T1=15;T2=10;A=30;Up=5;u=0.5;m=0.5;

[t,y]=ode45(@(t,y) zijian(t,y,b,Cl,Ct,u,w2,I,T1,T2,A,Up,m),[0 1],[0.5 0.5 0.5]);

points=1:1:length(t);

plot(t,y(:,3),'k:.','linewidth',1,'markersize',5,'markerfacecolor','r','markerindices',points);

hold on;

b=30;Cl=45;Ct=35;w2=20;I=30;T1=15;T2=10;A=30;Up=10;u=0.5;m=0.5;

[t,y]=ode45(@(t,y) zijian(t,y,b,Cl,Ct,u,w2,I,T1,T2,A,Up,m),[0 1],[0.5 0.5 0.5]);

points=1:1:length(t);

plot(t,y(:,3),'k-','linewidth',1,'markersize',5,'markerfacecolor','r','markerindices',points);

hold on;

b=30;Cl=45;Ct=35;w2=20;I=30;T1=15;T2=10;A=30;Up=15;u=0.5;m=0.5;

[t,y]=ode45(@(t,y) zijian(t,y,b,Cl,Ct,u,w2,I,T1,T2,A,Up,m),[0 1],[0.5 0.5 0.5]);

points=1:1:length(t);

plot(t,y(:,3),'k--','linewidth',1,'markersize',5,'markerfacecolor','r','markerindices',points);

hold on;

b=30;Cl=45;Ct=35;w2=20;I=30;T1=15;T2=10;A=30;Up=5;u=0.5;m=0.5;

[t,y]=ode45(@(t,y) zijian(t,y,b,Cl,Ct,u,w2,I,T1,T2,A,Up,m),[0 1],[0.5 0.5 0.8]);

points=1:1:length(t);

plot(t,y(:,3),'k:.','linewidth',1,'markersize',5,'markerfacecolor','r','markerindices',points);

hold on;

b=30;Cl=45;Ct=35;w2=20;I=30;T1=15;T2=10;A=30;Up=10;u=0.5;m=0.5;

[t,y]=ode45(@(t,y) zijian(t,y,b,Cl,Ct,u,w2,I,T1,T2,A,Up,m),[0 1],[0.5 0.5 0.8]);

points=1:1:length(t);

plot(t,y(:,3),'k-','linewidth',1,'markersize',5,'markerfacecolor','r','markerindices',points);

hold on;

b=30;Cl=45;Ct=35;w2=20;I=30;T1=15;T2=10;A=30;Up=15;u=0.5;m=0.5;

[t,y]=ode45(@(t,y) zijian(t,y,b,Cl,Ct,u,w2,I,T1,T2,A,Up,m),[0 1],[0.5 0.5 0.8]);

points=1:1:length(t);

plot(t,y(:,3),'k--','linewidth',1,'markersize',5,'markerfacecolor','r','markerindices',points);

hold on;

set(0,'defaultfigurecolor','w')

grid on

hold on

xlabel('$T$','interpreter','latex','Rotation',0);

ylabel('$Z$','interpreter','latex');

set(gca,'XTick',[0:0.2:1],'YTick',[0:0.2:1])

axis([0 1 0 1]);

legend('Up=5','Up=10','Up=15');
