# Supplementary material for: Strategies selection for building e-commerce platforms for agricultural wholesale markets: A tripartite evolutionary game perspective
Source: PLoS One. 2024 Jan 25;19(1):e0297360. doi: 10.1371/journal.pone.0297360 (PMC10810498; doi:10.1371/journal.pone.0297360)
Supplement: Attachment — Submitted filename: Response to Reviewers.pdf [file pone.0297360.s002.pdf]

November 24, 2023

Dear Editor and Reviewers,

Thank you for your letter and the reviewers' comments concerning our manuscript entitled "Research on the construction and promotion strategies of e-commerce platforms in agricultural wholesale markets" (ID: PONE-D-23-31750). Those comments are all valuable and very helpful for revising and improving our paper, as well as the important guiding significance to our researches. We have studied comments carefully and have made correction which we hope meet with approval. Below we explain how editor's and reviewers' comments were individually addressed,for easing the reviewing work,the blue text in the following passage is a quotation from the modified original text.

Response to the editor's comments

1. "The introduction section needs to provide more insight and primarily resolve the three key questions namely (a) what is the need of this research, (b) what are the research gaps identified by the authors, and (c) what is contribution of this manuscript in reference to academia and practice."

> **Our answer:** We have made revisions to the introduction section.This study primarily addresses the issue of how to build e-commerce platforms in agricultural wholesale markets scientifically. Through an analysis of previous studies, we found that there needs to be more in-depth exploration regarding the scientific construction of e-commerce platforms during the process of digital transformation in agricultural wholesale markets. Most literature focuses on the application of evolutionary game theory to study the digital transformation process of enterprises. At the same time, there is relatively little research on the key factor of how enterprises can build e-commerce platforms scientifically. Therefore, we determined the research theme of this paper. This study is beneficial in assisting agricultural wholesale markets to construct e-commerce platforms in a scientific manner and promoting the digital transformation of agricultural wholesale markets.

2. "The literature review segment can be further enhanced by creating two sub-headings namely (a) State of Agriculture Wholesale Market, and (b) promotional strategies of e-commerce platforms."

> **Our answer:** We have made revisions to the literature review section. We have made it clearer by introducing two subheadings. We have divided the literature review section into the following two parts (1) research on the e-commerce transformation of agricultural wholesale markets. (2) research on evolutionary game theory.

3. "The quality of images is poor. Kindly enhance the quality of the images."

> **Our answer:** We have processed the images again.

4. "As one of the reviewers observed, enhance the discussion section."

> **Our answer:** We have made further revisions to the article and strengthened the discussion section. In the original model, we only considered the case of agricultural

wholesale markets building their own e-commerce platforms. However, in the modified article, we analyze two behavioral choices for agricultural wholesale markets: building their own e-commerce platforms and using third-party e-commerce platforms. When agricultural wholesale markets lean towards building their own platforms, we discuss how to promote the construction of such platforms and encourage wholesalers to choose to use them. On the other hand, when agricultural wholesale markets are more inclined to use third-party platforms, we discuss how to promote their usage and encourage wholesalers to choose those platforms. Finally, we propose corresponding strategic recommendations from the perspectives of agricultural wholesale markets and the government.

5. "The referencing style is incorrect. Authors should adhere to the journal formatting guidelines and update the referencing style."

Our answer: We have made modifications to the citations in the references.

Special thanks to you for your good comments.

Review 1

R1.1 "The title does not provide a core theme of the topic"

Our answer: We have modified the title of the paper to Strategies selection for building e-commerce platforms for agricultural wholesale markets: A tripartite evolutionary game perspective. Agricultural wholesale markets have the option to build their own e-commerce platforms or by using third-party platforms. The main focus of this article is to discuss how agricultural wholesale markets can scientifically build e-commerce platforms and provide corresponding strategic recommendations through the application of evolutionary game model.

R1.2 " Please specify the source of the simulation data."

Our answer: We have added the data sources in the paper.The parameter assignments are as follows.$b = 30; C_l = 45; C_t = 35; w_2 = 20; I = 30; T_1 = 15; T_2 = 10; A = 30; U_p = 5; u = 0.5; m = 0.5$.The data assignments are based on the following.

(1)Reference[6] Yao XG. Government Interference Consumer Purchase Behavior and Brand Construction of Agricultural Products Based on Tripartite Evolutionary Game Theory. Journal of Agro-Forestry Economics and Management. 2023;22(171-181).

(2)Based on the actual situation, Building e-commerce platforms by agricultural wholesale markets themselves requires more costs compared to using third-party platforms, so we assume $C_l > C_t$.

(3)The parameters are simulated using MATLAB software, and the stable points of the system are found to be (0, 1, 0) and (1, 1, 1), which is consistent with the theoretical analysis. Therefore, the parameter assignments in this paper are considered reasonable.

R1.3 "The language of this manuscript needs help from native speakers."

Our answer: We apologize for the poor language of our manuscript. We worked on the manuscript for a long time and the repeated addition and removal of sentences and

sections obviously led to poor readability. We have now worked on both language and readability and have also involved native English speakers for language corrections. We really hope that the flow and language level have been substantially improved.

R1.4 "Please underscore the scientific value-added to your paper in your abstract. Your abstract should clearly state the essence of the problem you are addressing, what you did and what you found and recommend. That would help a prospective reader of the abstract to decide if they wish to read the entire article."

Our answer: We have revised the abstract. In the first half of the abstract, we provided background information, while in the middle part, we introduced the research methodology. In the final part, we presented the research findings and provided corresponding strategic recommendations. The modifications in the original text are as follows.

The rapid advancement and widespread implementation of digital technology have created opportunities for the e-commerce transformation of agricultural wholesale markets. The building of e-commerce platforms in this process is of utmost importance and should be approached methodically.This article analyzes the interests and behavioral choices of the agricultural wholesale markets, local government, and wholesalers by establishing a tripartite evolutionary game model. It applies replicator dynamics equations to describe the evolutionary strategies of each party. The findings of the study indicate that the behavioral choices of agricultural wholesale markets, local government, and wholesalers are influenced by their initial intentions. Furthermore, there exists a degree of alignment between the choices made by agricultural wholesale markets and wholesalers. The building of e-commerce platforms by agricultural wholesale markets can be facilitated through direct and indirect government subsidies; this also motivates wholesalers to adopt and utilize these platforms. Agricultural wholesale markets may further incentivize wholesalers to utilize their own e-commerce platforms by offering additional benefits. On the other hand, if the agricultural wholesale markets demonstrate strong initial inclinations toward using third-party e-commerce platforms. In this scenario, the local government has the potential to promote the widespread use of these platforms by providing both direct and indirect financial incentives to these markets, as well as actively encouraging wholesalers to participate in them. This study presents policy recommendations for agricultural wholesale markets and local government to support the effective implementation of e-commerce platforms in the agricultural wholesaler markets and facilitate a smooth transition to e-commerce in agricultural wholesale markets.

R1.5 " LINES31-49. This a very vague statement. These sentences do not provide any information on how the concept could be conceptualized? - The Introduction should have 1) a concise but complete justification of the topic's importance both academically and practically, and 2) an explanation of the gaps both in research and practice. Please review appropriate literature in the introduction, with the research question clearly arising from that review."

Our answer: We have made revisions to the article. In the concluding section of the introduction, we present(1) the theoretical contributions of this article. We highlight five key theoretical contributions.

(2)We also identify the gap between research and practice that exists in agricultural wholesale markets.We have found that there is still a lack of in-depth research on how to scientifically build e-commerce platforms in the process of agricultural wholesale market transformation towards e-commerce. In addition, most of the literature on the application of evolutionary game theory focuses on studying the e-commerce trans-

formation process of enterprises, with relatively less research on how enterprises can scientifically build e-commerce platforms, which is a critical factor. Therefore, we have identified this research topic for this paper.

Establishing e-commerce platforms is a crucial step toward the transformation of agricultural wholesale markets. Previous studies have focused only on where agricultural wholesale markets build their own e-commerce platforms. However, in reality, agricultural wholesale markets may also choose to use third-party platforms. Therefore, we modified the model to consider both scenarios. By analyzing the choices of agricultural wholesale markets between building their own e-commerce platforms and using third-party platforms, we aim to provide relevant policy recommendations for the scientific establishment of e-commerce platforms in agricultural wholesale markets. This research bridges the gap between theory and practice and provides targeted insights and guidance for the decision-making process of e-commerce transformation in agricultural wholesale markets.The modifications in the original text are as follows.

As conventional distribution firms, agricultural wholesale markets have proven essential in improving the connection between small producers and large markets;this is achieved through their architecture, rules, and mechanisms for price discovery. In China, it has been observed that agricultural wholesale markets have been observed to play a significant role in the distribution of products within rural areas, accounting for approximately 70% of the total distribution. Agricultural wholesale markets serve as a fundamental component of the fresh vegetable marketing system in Turkey. Similarly, in Bulgaria, approximately 75% of fishermen participate in the direct or indirect sale of their products through agricultural wholesale markets. The advent of e-commerce has led to the need for improved safety standards, transaction efficiency, responsiveness, information processing, and comprehensive services in agricultural wholesale markets due to the circulation of farm products. Therefore, in order to uphold the fundamental role of agricultural wholesale markets in agrarian circulation, it is imperative to undertake the restructuring of their business models. This endeavor is also crucial to industrial structural reform and advancement. From this standpoint, the promotion of e-commerce transformation and the upgrading of agricultural wholesale markets has emerged as an inevitable trend in accordance with the progress of the era. The extensive utilization of digital technology has yielded favorable outcomes for enterprises across multiple dimensions. First and foremost, enterprises have the capability to enhance the efficiency of their supply chain and decrease operational expenses by utilizing optimal data to inform decision-making processes, thus facilitating the formulation of comprehensive industrial growth strategies . Additionally, digital technology plays a crucial role in facilitating the e-commerce transformation of firms. Simultaneously, e-commerce platforms serve as a means for information exchange among wholesalers . Wholesale markets have effectively leveraged digital technology to their advantage by developing proprietary commodities trading platforms. When constructing e-commerce platforms for agricultural wholesale markets, there are two alternatives available: developing self-built platforms or utilizing third-party e-commerce platforms. When agricultural wholesale markets opt for self-building, they have the opportunity to tailor and create platforms that align with their specific requirements and distinctive attributes. The platforms have the capability to be created and tailored to meet the specific needs of market participants, offering a wide range of comprehensive features and services. Self-built platforms also give enhanced control over brand perception and user interaction, facilitating the provision of tailored services and solutions. Nevertheless, the development of self-built platforms necessitates a significant allocation of financial resources and technical expertise for their development and management. Additionally, these platforms may encounter difficulties in attracting a sufficient number of users during their early phase. By opting to utilize third-party platforms, agricultural wholesale

markets can expedite the process of selling and promoting their products by seamlessly integrating into pre-existing platforms. Moreover, third-party platforms typically offer sophisticated logistics and payment systems, thereby streamlining the supply chain management processes within agricultural wholesale markets. However, the utilization of third-party platforms may result in a lack of complete control over brand image and user experience. Additionally, the presence of advertisements and competing brands on these platforms might potentially influence sales outcomes. Hence, in the context of the e-commerce revolution within agricultural wholesale markets, the selection of suitable approaches for the systematic and rational build of e-commerce platforms emerges as a critical matter necessitating meticulous deliberation. To effectively tackle this issue, the present study formulates a three-party evolutionary game model that includes agricultural wholesale markets, local government, and wholesalers. It investigates the evolutionary dynamics of equilibrium strategies within the system under varying conditions and subsequently assesses the influence of relevant factors on system stability through numerical simulations. This study aims to answer the following questions.

1. How can the promotion of self-built e-commerce platforms in agricultural wholesale markets be enhanced, and what strategies may be employed to incentivize wholesalers to choose these platforms?

2. What strategies can be used to improve the promotion of third-party e-commerce platforms in agricultural wholesale markets and foster increased adoption among wholesalers?

This research study provides the following contributions: (1) This study aims to examine the process of e-commerce transformation in agricultural wholesale markets, with a specific focus on the crucial task of constructing e-commerce platforms. (2) This study aims to provide recommendations and insights for developing e-commerce platforms in agricultural wholesale markets by analyzing the options of self-built e-commerce platforms and utilizing third-party e-commerce platforms. (3) This study examines the impact of government subsidies on developing e-commerce platforms in agricultural wholesale markets. It aims to provide a scientific basis for the government to effectively implement policy measures that promote the construction of e-commerce platforms in agricultural wholesale markets. (4) This study examines the behavioral decisions made by agricultural wholesale markets, local government, and wholesalers, considering various original intents. (5) This study investigates the reciprocal impact of behavioral decisions and provides references and recommendations for developing e-commerce platforms in agricultural wholesale markets. The rest of this paper is structured as follows: The second part reviews existing research. The third part introduces the construction process of a tripartite evolutionary game model involving agricultural wholesale markets, local government, and wholesalers. The fourth part solves the game mode, explicitly examining the equilibrium strategies of each entity involved in constructing e-commerce platforms in agricultural wholesale markets. The fifth part conducts numerical simulations to analyze the behavioral choices of the three parties under different initial intentions and examines the key factors that influence their behavioral choices. The sixth and seventh parts summarize the research, offering pertinent suggestions for the scientific and rational construction of e-commerce platforms in agricultural wholesale markets and elaborating on this study's limitations and future research directions.
(Line 25-109)

R1.6 "LINES42-48.What authors wanted to convey. Here author must build research gap following the previous studies.-The manuscript does not answer the following concerns: Why is it timeliness to explore such a study? What makes this study different from the previously published studies?

Are there any similarly findings in line with the previously published studies? Are the findings different from prior academic studies that were conducted elsewhere, if any? What it requires, what are the new technologies, some recent issue highlights the importance. "

Our answer: We have made revisions to the manuscript. In the introduction and literature review sections, we have addressed this issue. –Why is it timeliness to explore such a study? We have made revisions to the article. In the introduction section, we emphasize the necessity of addressing this question in detail. Building e-commerce platforms in the process of transforming agricultural wholesale markets into e-commerce is a crucial step. In this process, agricultural wholesale markets can either build their own e-commerce platforms or use third-party e-commerce platforms. By analyzing these two scenarios, this article presents corresponding policy recommendations for the scientific establishment of e-commerce platforms in agricultural wholesale markets.The modifications in the original text are as follows.

Additionally, digital technology plays a crucial role in facilitating the e-commerce transformation of firms. Simultaneously, e-commerce platforms serve as a means for information exchange among wholesalers. Wholesale markets have effectively leveraged digital technology to their advantage by developing proprietary commodities trading platforms. When constructing e-commerce platforms for agricultural wholesale markets, there are two alternatives available: developing self-built platforms or utilizing third-party e-commerce platforms. When agricultural wholesale markets opt for self-building, they have the opportunity to tailor and create platforms that align with their specific requirements and distinctive attributes. The platforms have the capability to be created and tailored to meet the specific needs of market participants, offering a wide range of comprehensive features and services. Self-built platforms also give enhanced control over brand perception and user interaction, facilitating the provision of tailored services and solutions. Nevertheless, the development of self-built platforms necessitates a significant allocation of financial resources and technical expertise for their development and management. Additionally, these platforms may encounter difficulties in attracting a sufficient number of users during their early phase. By opting to utilize third-party platforms, agricultural wholesale markets can expedite the process of selling and promoting their products by seamlessly integrating into pre-existing platforms. Moreover, third-party platforms typically offer sophisticated logistics and payment systems, thereby streamlining the supply chain management processes within agricultural wholesale markets. However, the utilization of third-party platforms may result in a lack of complete control over brand image and user experience. Additionally, the presence of advertisements and competing brands on these platforms might potentially influence sales outcomes. Hence, in the context of the e-commerce revolution within agricultural wholesale markets, the selection of suitable approaches for the systematic and rational build of e-commerce platforms emerges as a critical matter necessitating meticulous deliberation. To effectively tackle this issue, the present study formulates a three-party evolutionary game model that includes agricultural wholesale markets, local government, and wholesalers. It investigates the evolutionary dynamics of equilibrium strategies within the system under varying conditions and subsequently assesses the influence of relevant factors on system stability through numerical simulations. This study aims to answer the following questions.
(Line 46-78)

–What makes this study different from the previously published studies? Are there any similarly findings in line with the previously published studies? Are the findings different from prior academic studies that were conducted elsewhere, if any? In the literature review section, we provide a detailed overview of previous research and point out their shortcomings - the current research needs more in-depth exploration regarding

how to scientifically build e-commerce platforms in the process of transforming agricultural wholesale markets. In terms of the application of evolutionary game theory, most literature focuses on using this theory to study the e-commerce transformation process of enterprises, but there needs to be more research on how to build e-commerce platforms in this critical factor scientifically. Identifying this gap further underscores the necessity of our study.The modifications in the original text are as follows.

Presently, experts from both domestic and international contexts have unequivocally acknowledged the crucial significance of e-commerce technology in facilitating the transformative process of agricultural wholesale markets within the realm of electronic commerce. The facilitation of e-commerce transformation in agricultural wholesale markets is achievable through various means, such as improving infrastructure and implementing supportive government policies. However, it is imperative to conduct a comprehensive study to ascertain the scientific methods for building e-commerce platforms during the process of transforming agricultural wholesale markets into e-commerce. Furthermore, the majority of existing research primarily focuses on examining the use of evolutionary game theory in analyzing the process of e-commerce transformation undertaken by firms. In contrast, there is limited research on firms' scientific build of e-commerce platforms. Hence, this study employs evolutionary game theory as a framework to examine the trajectory of agricultural wholesale markets in adopting e-commerce platforms, taking into account various initial intentions. Additionally, this research offers relevant recommendations to facilitate the transformation of agricultural wholesale markets into e-commerce entities.
(Line 196-211)

R1.7 "There is no flow in the text. It depends on the lack of proofreading but also on the fact that many statements and claims are made without being followed up by a clear and logical discussion. It is especially problematic in the introduction that brings up a number of findings from different areas without linking them together. "

Our answer: We have reorganized and proofread the main body of the text.

R1.8 " More importantly, the choice of the questionnaire questions should be explained in light of the theory and the prior literature on the topic. The arguments are simply relationships and causes very close to the replication of many studies dealing with the same thing."

Our answer: In this paper, we combine theory and previous relevant literature to explain the relevant issues. Our argument is based on existing research, focusing on exploring the relationships and causal relationships between issues. At the same time, we choose the model research method as the main method and explain the construction process of the model in detail in the thesis while also explaining the rationality of parameter assignment. Through this model research, we have proposed corresponding policy recommendations from the perspective of agricultural wholesale markets and local government, aiming to help better agricultural wholesale markets smoothly build e-commerce platforms and promote the e-commerce transformation of agricultural wholesale markets. In addition, our research also verifies some of the problems in the process of building e-commerce platforms for agricultural wholesale markets, such as insufficient funding, imperfect policies, and difficult-to-change purchasing and selling habits of wholesalers (referenced literature 2, 3, 5 [5][2][3]). These empirical results further support our research conclusions.

R1.9 "Methodology: Model.. I suggest authors here build your main heading on research and data methodology. Clearly explain the model building process, and what previous studies have used similar models."

Our answer: Thank you very much for your suggestion. In the title, we have clearly stated the method we used, the Tripartite Evolutionary Game Model. We have also modified the title: "Strategies selection for building e-commerce platforms for agricultural wholesale markets: A tripartite evolutionary game perspective." In the main text, we have provided a detailed explanation of the model construction process in the third section. In the fourth section, we have categorized the analysis using second-level and third-level headings.

R1.10 "The authors should emphasize the important role of digital technology in future research."

Our answer: In the introduction section, we have incorporated the role of digital technology. The modifications in the original text are as follows.

The extensive utilization of digital technology has yielded favorable outcomes for enterprises across multiple dimensions. First and foremost, enterprises have the capability to enhance the efficiency of their supply chain and decrease operational expenses by utilizing optimal data to inform decision-making processes, thus facilitating the formulation of comprehensive industrial growth strategies. Additionally, digital technology plays a crucial role in facilitating the e-commerce transformation of firms. Simultaneously, e-commerce platforms serve as a means for information exchange among wholesalers. Wholesale markets have effectively leveraged digital technology to their advantage by developing proprietary commodities trading platforms.
(Line 42-50)

Additionally, we have included the role of digital technology in the discussion section as well. The modifications in the original text are as follows.

Meanwhile, the evolutionary results of the game system also demonstrate that the widespread application of digital technologies (artificial intelligence, blockchain, cloud computing, and digital platforms) has provided corresponding technological conditions for the construction of agricultural e-commerce platforms in wholesale markets.
(Line 681-684)

R1.11 "11.Please consider this structure for the manuscript final part. -Discussion -Conclusion -Managerial Implication -Practical/Social Implications"

Our answer: In the sixth section, which is the discussion section. In the seventh section, we have also divided into three subsections: the first subsection is the conclusion, the second subsection is the recommendations, and the third subsection is the contributions and limitations.

We have made the following modifications to the sixth and seventh sections. We have supplemented the relevant parts in the paper.The modifications in the original text are as follows.

6.Discussion

Comparing the stability analysis and simulation results of the game model, it is found that the agricultural wholesale markets, local government, and wholesalers consistently maintain stable strategies in the simulation analysis. During the simulation process, the influence of the initial intentions of the agricultural wholesale markets, local government, and wholesalers on each other's behavioral choices is considered. Variables such as direct subsidies, indirect subsidies, synergistic benefits, and additional profits are introduced. Due to the influence of environmental and decision-making uncer-

tainties on the strategic evolution of the three parties, the decision-making intentions fluctuate randomly. However, the overall trend of the simulation results is consistent with the stability analysis results, demonstrating the validity of this research method.

Meanwhile, the evolutionary results of the game system also demonstrate that the widespread application of digital technologies (artificial intelligence, blockchain, cloud computing, and digital platforms) has provided corresponding technological conditions for the construction of agricultural e-commerce platforms in wholesale markets. However, there are still problems with insufficient funding, inadequate policies, and difficulty in changing purchasing and sales habits among wholesalers during the construction process of these platforms. Therefore, the use of evolutionary game models and simulations is effective.

7.Conclusion

7.1Research conclusions

(1)In the game system comprising agricultural wholesale markets, local government, and wholesalers, the behavioral choices of agricultural wholesale markets are influenced by the initial intentions of the local government and wholesalers. When agricultural wholesale markets have a higher initial intention to choose self-built e-commerce platforms, increased subsidies from the local government and higher intentions of wholesalers to use agricultural wholesale markets' self-built e-commerce platforms are conducive to promoting the use of self-built e-commerce platforms in agricultural wholesale markets. When agricultural wholesale markets show a higher intention to choose third-party e-commerce platforms, increased subsidies from the local government and a stronger intention among wholesalers to utilize these platforms can be beneficial for agricultural wholesale markets to use third-party e-commerce platforms. The local government is the main entity exerting influence, and the behavioral choices of agricultural wholesale markets and wholesalers can not change the behavioral choices of the local government. The behavioral choices of agricultural wholesale markets and the local government influence the behavioral choices of wholesalers. However, the behavioral choices of wholesalers tend to exhibit strong inertia. Once they form their usage habits, it becomes challenging for external entities to alter their behavioral choices.

(2)The behavioral choices of agricultural wholesale markets and wholesalers have a certain level of synchronization. As the mutual benefits of their actions increase, the synchronicity of their behavioral strategies is also enhanced. If agricultural wholesale markets have a strong preference for creating their own e-commerce platforms, providing direct subsidies from the local government to these markets will encourage the development of such platforms. These subsidies will also encourage wholesalers to use the self-built e-commerce platforms of agricultural wholesale markets. Indirect subsidies from the local government to agricultural wholesale markets that choose self-built e-commerce platforms also encourage the adoption of these platforms and facilitate wholesalers in their decision to use them. The gradual increase in additional benefits for wholesalers who utilize self-built e-commerce platforms in agricultural wholesale markets is beneficial for promoting these options.

(3)When agricultural wholesale markets initially show a strong preference for third-party e-commerce platforms, direct subsidies from the local government can encourage the adoption of third-party e-commerce platforms by agricultural wholesale markets. Additionally, these subsidies also benefit the promotion of wholesalers' choice of third-party e-commerce platforms. Indirect subsidies from the local government to agricultural wholesale markets that choose third-party e-commerce platforms also encourage

the adoption of these platforms by agricultural wholesale markets and facilitate the use of third-party e-commerce platforms by wholesalers.

7.2Policy recommendations

(1)As an essential policy maker, the government can provide construction fund subsidies to agricultural wholesale markets that choose to build their own e-commerce platforms through fiscal budget allocations. This will encourage and support the development of self-built e-commerce platforms by agricultural wholesale markets. Additionally, the government can reduce the costs of building agricultural wholesale markets' self-built e-commerce platforms through monetary supply and interest rate regulation. For instance, lowering loan interest rates can further promote the establishment of self-built e-commerce platforms by agricultural wholesale markets. When agricultural wholesale markets choose to use third-party e-commerce platforms, the government can directly subsidize the platform service fees, which cover maintenance, technical support, and promotion, thus reducing the financial burden for agricultural wholesale markets. Furthermore, the government can help reduce the resources, human resources, and management expenses required for using third-party e-commerce platforms by providing low-interest loans and other supportive measures.

(2)Agricultural wholesale markets and wholesalers' behavior choices are somewhat synchronized. When agricultural wholesale markets choose to build their own e-commerce platforms, they can gather information about the specific requirements of wholesalers through face-to-face interactions and online research. By utilizing data analysis tools to analyze wholesalers' behavioral data and behavior trajectories, agricultural wholesale markets can gain insights into wholesalers' search and purchase preferences and identify any problems they may encounter when using the platforms. This insight can help agricultural wholesale markets better understand the needs of wholesalers who are willing to use self-built e-commerce platforms in agricultural wholesale markets which can promote the use of such platforms by wholesalers. Moreover, agricultural wholesale markets can customize their independent and self-built e-commerce platforms according to the needs and preferences of wholesalers. This allows them to provide better user experiences and enhance their competitiveness in the market,and also makes it easier for wholesalers to browse and purchase products, enjoy personalized services, and obtain accurate product information, thereby enhancing wholesalers' satisfaction and generating additional revenue. When agricultural wholesale markets choose to use third-party platforms, they can actively participate in various activities organized by the platforms to create a tight marketing atmosphere with other merchants. This can promote the use of the platforms by wholesalers who are willing to use third-party e-commerce platforms.

7.3Contributions and limitations

This research makes significant contributions to both theoretical knowledge and management practices. Firstly, the research findings reveal the initial intentions of agricultural wholesale markets, local government, and wholesalers regarding each other's behavioral choices, which helps to deepen the understanding of the strategic decision-making and interaction processes among the different participants in the tripartite evolutionary game environment. Secondly, the research proposes policy recommendations for building e-commerce platforms for agricultural wholesale markets by analyzing government subsidies, collaborative benefits, and additional benefits. In particular, government subsidies and collaborative benefits serve as important influencing factors, and it is recommended that the government increase funding to support and cooperation efforts for agricultural wholesale markets to promote their development towards

e-commerce. Furthermore, while it may be challenging to attain additional benefits directly, it is advisable for agricultural wholesale markets to enhance their internal innovation capabilities, optimize product supply, and improve service quality to enhance market competitiveness and attractiveness. This research provides valuable experiences and insights for transforming agricultural wholesale markets in e-commerce. It is also a reference and foundation for policy formulation and management practices. However, this research also has certain limitations. During the construction of the game model, only the mutual influence between agricultural wholesale markets, local government, and wholesalers was considered. Other aspects, such as logistics during the construction of e-commerce platforms for agricultural wholesale markets, should have been considered. Therefore, future research can further advance the construction process of e-commerce platforms for agricultural wholesale markets by considering more participating entities, thus promoting the e-commerce transformation process more effectively.
(Line 670-789)

R1.12 "Please make sure your conclusions section underscores the scientific value-added of your paper, and/or the applicability of your findings/results."

Our answer: We have supplemented relevant parts in the thesis. The sixth part is the discussion part. In the discussion part, we detailed the applicability of the results by referring to previous research. By simulating the results, we found that the overall trend of the simulation results and the stability analysis results are consistent, which indicates that the establishment of the model has a certain validity. We also related it to previous research: this study confirms the problems of insufficient funding, imperfect policies, and difficult-to-change purchasing and selling habits of wholesalers in the process of building e-commerce platforms for agricultural wholesale markets. Also, it demonstrates the scientific value of our results.The modifications in the original text are as follows.

Comparing the stability analysis and simulation results of the game model, it is found that the agricultural wholesale markets, local government, and wholesalers consistently maintain stable strategies in the simulation analysis. During the simulation process, the influence of the initial intentions of the agricultural wholesale markets, local government, and wholesalers on each other's behavioral choices is considered. Variables such as direct subsidies, indirect subsidies, synergistic benefits, and additional profits are introduced. Due to the influence of environmental and decision-making uncertainties on the strategic evolution of the three parties, the decision-making intentions fluctuate randomly. However, the overall trend of the simulation results is consistent with the stability analysis results, demonstrating the validity of this research method. Meanwhile, the evolutionary results of the game system also demonstrate that the widespread application of digital technologies (artificial intelligence, blockchain, cloud computing, and digital platforms) has provided corresponding technological conditions for the construction of agricultural e-commerce platforms in wholesale markets. However, there are still problems with insufficient funding, inadequate policies, and difficulty in changing purchasing and sales habits among wholesalers during the construction process of these platforms. Therefore, the use of evolutionary game models and simulations is effective.
(Line 670-688)

Special thanks to you for your good comments.

Review 2

R2.1 "In line 8-11, the abstract points out that "The research findings indicate that government subsidies play a key role in the establishment of e-commerce platforms in agricultural wholesale markets. When government subsidies are sufficient, the agricultural wholesale markets are more willing to choose the establishment of e-commerce platforms." In reality, agricultural products are mostly sold on various e-commerce platforms, and the proportion of self-established e-commerce platforms is not high. The wholesale market for agricultural products mainly exists offline, and its main role is to provide a market for agricultural product trading. The establishment of agricultural product e-commerce platforms focuses more on the operation and maintenance of e-commerce platforms. The durability of government subsidies is an important consideration."

Our answer: We indeed overlooked this aspect. In the original model, we only considered the scenario where agricultural wholesale markets choose to build their own e-commerce platforms. However, in reality, agricultural wholesale markets also have the option to use third-party e-commerce platforms. We have made modifications to the model in the paper to address this. In the new model, we have separately considered the cases where agricultural wholesale markets have a greater initial inclination to build their own e-commerce platforms or use third-party e-commerce platforms. We have provided corresponding policy recommendations for scientifically constructing e-commerce platforms for agricultural wholesale markets and promoting their e-commerce transformation. The modifications in the original text are as follows.

The behavioral choices of agricultural wholesale markets and wholesalers have a certain level of synchronization. As the mutual benefits of their actions increase, the synchronicity of their behavioral strategies is also enhanced. If agricultural wholesale markets have a strong preference for creating their own e-commerce platforms, providing direct subsidies from the local government to these markets will encourage the development of such platforms. These subsidies will also encourage wholesalers to use the self-built e-commerce platforms of agricultural wholesale markets. Indirect subsidies from the local government to agricultural wholesale markets that choose self-built e-commerce platforms also encourage the adoption of these platforms and facilitate wholesalers in their decision to use them. The gradual increase in additional benefits for wholesalers who utilize self-built e-commerce platforms in agricultural wholesale markets is beneficial for promoting these options.
(Line 709-720)

In the conclusion and recommendation section of this paper, we propose suggestions from the perspectives of agricultural wholesale markets and local government, providing a theoretical basis for the scientific construction of e-commerce platforms in agricultural wholesale markets.The modifications in the original text are as follows.

7.Conclusion

7.1Research conclusions

(1)In the game system comprising agricultural wholesale markets, local government, and wholesalers, the behavioral choices of agricultural wholesale markets are influenced by the initial intentions of the local government and wholesalers. When agricultural wholesale markets have a higher initial intention to choose self-built e-commerce platforms, increased subsidies from the local government and higher intentions of wholesalers to use agricultural wholesale markets' self-built e-commerce platforms are conducive to promoting the use of self-built e-commerce platforms in agricultural wholesale markets. When agricultural wholesale markets show a higher intention to choose third-party e-commerce platforms, increased subsidies from the local government and a stronger intention among wholesalers to utilize these platforms can be beneficial for

agricultural wholesale markets to use third-party e-commerce platforms. The local government is the main entity exerting influence, and the behavioral choices of agricultural wholesale markets and wholesalers can not change the behavioral choices of the local government. The behavioral choices of agricultural wholesale markets and the local government influence the behavioral choices of wholesalers. However, the behavioral choices of wholesalers tend to exhibit strong inertia. Once they form their usage habits, it becomes challenging for external entities to alter their behavioral choices.

(2)The behavioral choices of agricultural wholesale markets and wholesalers have a certain level of synchronization. As the mutual benefits of their actions increase, the synchronicity of their behavioral strategies is also enhanced. If agricultural wholesale markets have a strong preference for creating their own e-commerce platforms, providing direct subsidies from the local government to these markets will encourage the development of such platforms. These subsidies will also encourage wholesalers to use the self-built e-commerce platforms of agricultural wholesale markets. Indirect subsidies from the local government to agricultural wholesale markets that choose self-built e-commerce platforms also encourage the adoption of these platforms and facilitate wholesalers in their decision to use them. The gradual increase in additional benefits for wholesalers who utilize self-built e-commerce platforms in agricultural wholesale markets is beneficial for promoting these options.

(3)When agricultural wholesale markets initially show a strong preference for third-party e-commerce platforms, direct subsidies from the local government can encourage the adoption of third-party e-commerce platforms by agricultural wholesale markets. Additionally, these subsidies also benefit the promotion of wholesalers' choice of third-party e-commerce platforms. Indirect subsidies from the local government to agricultural wholesale markets that choose third-party e-commerce platforms also encourage the adoption of these platforms by agricultural wholesale markets and facilitate the use of third-party e-commerce platforms by wholesalers.

7.2Policy recommendations

(1)As an essential policy maker, the government can provide construction fund subsidies to agricultural wholesale markets that choose to build their own e-commerce platforms through fiscal budget allocations. This will encourage and support the development of self-built e-commerce platforms by agricultural wholesale markets. Additionally, the government can reduce the costs of building agricultural wholesale markets' self-built e-commerce platforms through monetary supply and interest rate regulation. For instance, lowering loan interest rates can further promote the establishment of self-built e-commerce platforms by agricultural wholesale markets. When agricultural wholesale markets choose to use third-party e-commerce platforms, the government can directly subsidize the platform service fees, which cover maintenance, technical support, and promotion, thus reducing the financial burden for agricultural wholesale markets. Furthermore, the government can help reduce the resources, human resources, and management expenses required for using third-party e-commerce platforms by providing low-interest loans and other supportive measures.

(2)Agricultural wholesale markets and wholesalers' behavior choices are somewhat synchronized. When agricultural wholesale markets choose to build their own e-commerce platforms, they can gather information about the specific requirements of wholesalers through face-to-face interactions and online research. By utilizing data analysis tools to analyze wholesalers' behavioral data and behavior trajectories, agricultural wholesale markets can gain insights into wholesalers' search and purchase preferences and identify any problems they may encounter when using the platforms. This insight can

help agricultural wholesale markets better understand the needs of wholesalers who are willing to use self-built e-commerce platforms in agricultural wholesale markets which can promote the use of such platforms by wholesalers. Moreover, agricultural wholesale markets can customize their independent and self-built e-commerce platforms according to the needs and preferences of wholesalers. This allows them to provide better user experiences and enhance their competitiveness in the market,and also makes it easier for wholesalers to browse and purchase products, enjoy personalized services, and obtain accurate product information, thereby enhancing wholesalers' satisfaction and generating additional revenue. When agricultural wholesale markets choose to use third-party platforms, they can actively participate in various activities organized by the platforms to create a tight marketing atmosphere with other merchants. This can promote the use of the platforms by wholesalers who are willing to use third-party e-commerce platforms.
(Line 689-763)

R2.2 "In introduction, this paper fails to elaborate on the necessity of building a self-built e-commerce platform for agricultural product wholesale markets. which is not convincing."

Our answer: We have made modifications to the model. In the introduction section, we separately analyze the decision-making process of agricultural wholesale markets in constructing e-commerce platforms, considering the options of building their own platforms or using third-party platforms. We also analyze the advantages and disadvantages of these two platform construction methods. If agricultural wholesale markets choose to build their own e-commerce platforms, they can customize and develop platforms that suit their own needs and characteristics.This kind of platform can be designed and tailored based on the requirements of market participants, providing comprehensive functions and services. Building their own platforms also allows better control over brand images and user experiences, as well as offering personalized services and solutions. However, building platforms require significant investment in funds and technical resources for development, operation, and maintenance and may face challenges in the early stages with a limited user base. On the other hand, if they choose to use third-party platforms, agricultural wholesale markets can quickly start selling and promoting products by accessing existing platforms. In addition, third-party platforms usually provide well-established logistics and payment systems, simplifying supply chain management for agricultural wholesale markets. However, using third-party platforms may result in a lack of full control over brand images and user experiences, and advertisements and competing brands on the platform may have some influence on sales. By analyzing the pros and cons of the two behavioral choices during the construction of e-commerce platforms for agricultural wholesale markets and identifying action plans for different platform construction paths, we provide policy recommendations for scientifically constructing e-commerce platforms for agricultural wholesale markets.The modifications in the original text are as follows.

The extensive utilization of digital technology has yielded favorable outcomes for enterprises across multiple dimensions. First and foremost, enterprises have the capability to enhance the efficiency of their supply chain and decrease operational expenses by utilizing optimal data to inform decision-making processes, thus facilitating the formulation of comprehensive industrial growth strategies. Additionally, digital technology plays a crucial role in facilitating the e-commerce transformation of firms. Simultaneously, e-commerce platforms serve as a means for information exchange among wholesalers. Wholesale markets have effectively leveraged digital technology to their advantage by developing proprietary commodities trading platforms. When constructing e-commerce platforms for agricultural wholesale markets, there are two alternatives available: developing self-built platforms or utilizing third-party e-commerce platforms. When agri-

cultural wholesale markets opt for self-building, they have the opportunity to tailor and create platforms that align with their specific requirements and distinctive attributes. The platforms have the capability to be created and tailored to meet the specific needs of market participants, offering a wide range of comprehensive features and services. Self-built platforms also give enhanced control over brand perception and user interaction, facilitating the provision of tailored services and solutions. Nevertheless, the development of self-built platforms necessitates a significant allocation of financial resources and technical expertise for their development and management. Additionally, these platforms may encounter difficulties in attracting a sufficient number of users during their early phase. By opting to utilize third-party platforms, agricultural wholesale markets can expedite the process of selling and promoting their products by seamlessly integrating into pre-existing platforms. Moreover, third-party platforms typically offer sophisticated logistics and payment systems, thereby streamlining the supply chain management processes within agricultural wholesale markets. However, the utilization of third-party platforms may result in a lack of complete control over brand image and user experience. Additionally, the presence of advertisements and competing brands on these platforms might potentially influence sales outcomes. Hence, in the context of the e-commerce revolution within agricultural wholesale markets, the selection of suitable approaches for the systematic and rational build of e-commerce platforms emerges as a critical matter necessitating meticulous deliberation. To effectively tackle this issue, the present study formulates a three-party evolutionary game model that includes agricultural wholesale markets, local government, and wholesalers. It investigates the evolutionary dynamics of equilibrium strategies within the system under varying conditions and subsequently assesses the influence of relevant factors on system stability through numerical simulations. This study aims to answer the following questions.

1. How can the promotion of self-built e-commerce platforms in agricultural wholesale markets be enhanced, and what strategies may be employed to incentivize wholesalers to choose these platforms?

2. What strategies can be used to improve the promotion of third-party e-commerce platforms in agricultural wholesale markets and foster increased adoption among wholesalers?
(Line 42-84)

R2.3 "In literature review, rarely discusses the application of system dynamics principles in the selection of government subsidy strategies."

Our answer: We have revised the text regarding this issue. In the literature review section, we have added relevant literature.The modifications in the original text are as follows.

Evolutionary game models and their applications

Traditional game theory posits that individuals are "completely rational" decision-makers.However, in reality, decision-makers can only make decisions that align with their expectations rather than optimal decisions due to limitations in their cognitive abilities and differences in environmental information. The behavior adjustment by game participants should be viewed as a dynamic evolutionary process, which forms the essence of how evolutionary game theory surpasses the assumption of complete rationality. Emphasizing dynamic equilibrium, evolutionary game theory can demonstrate the dynamic evolutionary process over time, helping to identify optimal strategies and address the actors' incomplete rationality. This theoretical perspective has found extensive applications in supply chain management and the study of the evolution of

system structures. By constructing an evolutionary game model among enterprises, governments, and digital technology platforms, Tang et al. suggested that enterprises must ensure their profitability and efficient operation during the e-commerce transformation and upgrading. Dong et al. utilized an evolutionary game model to examine the impact of government subsidies on the digital transformation process of enterprises and proposed measures such as enhancing digital infrastructure construction, establishing a dynamic subsidy adjustment mechanism, and building a collaborative and synergistic digital ecosystem. Sun et al. utilized dual-population evolutionary game theory to investigate the evolutionary process and influencing factors of the cooperation mechanism in the pork supply chain and devise a series of measures to promote effective competition and cooperation within the high-quality pork supply chain. These measures include reducing cooperation costs, increasing cooperation income, and establishing a fair and efficient system for income distribution and risk compensation.Through the analysis of an evolutionary game model, Lin et al. discovered that the benefits of digital technology empowerment, spillover effects, and supervisory effects exhibit positive correlations with governments, platform centers, and node enterprises' willingness to undergo e-commerce transformation.
(Line 166-194)

R2.4 "In Game process analysis, line 129-131, this paper assumed that "In order to encourage the agricultural wholesale markets to establish e-commerce platforms, the government will provide subsidies to the markets." In reality, more professional platform providers establish and operate e-commerce platforms than wholesale markets for agricultural products. This assumption seems to be inconsistent with reality."

Our answer: This is indeed something we overlooked. We have made modifications to the article regarding this issue. We have separately discussed the situations where agricultural wholesale markets have a high initial willingness to choose either self-built e-commerce platforms or third-party e-commerce platforms. In the process of transforming agricultural wholesale markets into e-commerce, the government will provide direct and indirect subsidies to those agricultural wholesale markets that choose to establish e-commerce platforms. When agricultural wholesale markets choose to build their own e-commerce platforms, the government will provide direct and indirect subsidies to them. The direct subsidy proportion from the local government is denoted as 'u,' and the indirect subsidy proportion is denoted as '1-m'. When agricultural wholesale markets choose to use third-party platforms, the local government will provide direct subsidies of '1-u' and indirect subsidies of 'm'. In the modified article, the subsidies from the local government to the agricultural wholesale markets are intended to promote the establishment of better e-commerce platforms and facilitate the transformation process towards e-commerce.The modifications in the original text are as follows.

The local government designates the direct subsidy amount as variable I, while the allocation coefficient is represented by variable U. When agricultural wholesale markets choose to build their own e-commerce platforms, they usually encounter expenses related to technology development and maintenance and costs associated with human resources. Conversely, agricultural wholesale markets that decide to utilize third-party platforms will receive direct subsidies of $(1-u)I$ from the local government. When agricultural wholesale markets choose to build their own e-commerce platforms, the cost that the markets have to bear is represented by m. This means that the indirect subsidy provided by the local government for the agricultural wholesale markets to build their own e-commerce platforms is $1-m$. On the other hand, when agricultural wholesale markets choose to use third-party e-commerce platforms, the cost that the markets have to bear is $1-m$, and the indirect subsidy provided by the local

government for the agricultural wholesale markets using third-party platforms is m. (Line 260-270)

R2.5 "In Parameter settings, line 158-160, this paper argued that "The additional profits gained from establishing e-commerce platforms include benefits derived from the platforms' advantages in quick supply-demand matching, information transmission, and capital support." But this is not the case in reality. The cultivation and promotion of any e-commerce platform may incur huge sunk costs."

Our answer: This is indeed something we overlooked. In the revised version of the article, we believe that agricultural wholesale markets can either build their own e-commerce platforms or use third-party platforms. Since the cost of building their own e-commerce platforms is higher than choosing to use third-party platforms, we assume thatC_l(cost of building own platforms) is greater than Ct (cost of using third-party platforms).The modifications in the original text are as follows.

When agricultural wholesale markets want to build their own e-commerce platforms, they commonly encounter charges related to technology development, maintenance, human resource expenditures, and other associated costs designated as C_l. However, when agricultural wholesale markets want to utilize third-party e-commerce platforms, they typically incur expenses associated with the management and operation of e-commerce enterprises, referred to as C_t.
(Line 249-254)

R2.6 "In Parameter settings, line 172-173, this paper pointed out that "The government's adoption of subsidy measures can enhance confidence and generate positive social reputation effects, denoted as A." How to measure A?"

Our answer: We have redefined A in the article and changed the game entity 'government' to 'local government'. A refers to the reward received by the local government if it chooses to provide subsidies. The reference for this assumption can be found in the following literature. [4]

R2.7 "The biggest problem in constructing Payoff Matrix based on system dynamics principles is relative idealization, which makes it difficult to consider multiple factors. This article fails to solve this problem"

Our answer: The evolutionary game model has certain limitations inherent in the model itself. In order to overcome these limitations, we have made revisions to the original model, taking into consideration the two options available to agricultural wholesale markets in order to promote the transformation towards e-commerce: building their own platforms or using third-party platforms. Additionally, we have introduced more parameters to consider the impact of direct subsidies, indirect subsidies, collaborative benefits, and additional benefits on the evolution of the system. Furthermore, we have conducted simulations under basic scenarios to demonstrate that the simulation results align with the theoretical analysis, thus validating the effectiveness of the analysis.In the discussion section, we have compared our results with previous literature, providing evidence of their reliability. We have also presented scientifically sound recommendations for the establishment of e-commerce platforms in agricultural wholesale markets to promote the transformation towards e-commerce. The article provides the following supplementary information regarding this issue.

Supplement 1

While the initial values of the parameters do have some impact on the rate and scope of their evolution, they do not alter the overall patterns and outcomes. The accuracy and practicality of the model are essential considerations in the process of establishing a model. Hence, in accordance with the model's criteria and drawing upon previous studies[6][1], alongside considering the practical circumstances, it is unavoidable that choosing to build proprietary platforms within agricultural wholesale markets will lead to higher expenses compared to using third-party platforms. The assigned values for each parameter are as follows:$b = 30; Cl = 45; Ct = 35; w2 = 20; I = 30; T1 = 15; T2 = 10; A = 30; Up = 5; u = 0.5; m = 0.5$. This study also conducted a basic situation simulation, and the simulation results are as follows.

From Figure 1, it can be observed that the system has two equilibrium points at this time, namely (0, 1, 0) and (1, 1, 1), which represent the behavioral choices of each party as follows: the first equilibrium point illustrates the choices of the agricultural wholesale markets using third-party platforms, local government subsidies, and wholesalers using third-party e-commerce platforms. The second equilibrium point represents the agricultural wholesale markets' choices to build their own e-commerce platforms, local government subsidies, and wholesalers using the agricultural wholesale markets' e-commerce platforms. These choices are consistent with the theoretical analysis mentioned earlier. Therefore, this parameter assignment has a certain level of validity.
(Line 363-385)

Supplement 2

Comparing the stability analysis and simulation results of the game model, it is found that the agricultural wholesale markets, local government, and wholesalers consistently maintain stable strategies in the simulation analysis. During the simulation process, the influence of the initial intentions of the agricultural wholesale markets, local government, and wholesalers on each other's behavioral choices is considered. Variables such as direct subsidies, indirect subsidies, synergistic benefits, and additional profits are introduced. Due to the influence of environmental and decision-making uncertainties on the strategic evolution of the three parties, the decision-making intentions fluctuate randomly. However, the overall trend of the simulation results is consistent with the stability analysis results, demonstrating the validity of this research method.

Meanwhile, the evolutionary results of the game system also demonstrate that the widespread application of digital technologies (artificial intelligence, blockchain, cloud computing, and digital platforms) has provided corresponding technological conditions for the construction of agricultural e-commerce platforms in wholesale markets. However, there are still problems with insufficient funding[5], inadequate policies[2], and difficulty in changing purchasing and sales habits among wholesalers during the construction process of these platforms[3]. Therefore, the use of evolutionary game models and simulations is effective.
(Line 671-688)

R2.8 "The Payoff Matrix constructed in the paper has several possibilities, but it is only a theoretical comparison. The practical significance is relatively small."

Our answer:
1.Constructing the payoff matrix is a common tool aimed at providing a structured and clear way to compare the advantages and disadvantages of different decisions or strategies. Although this is just a theoretical comparison, it can provide insights and inspirations to help decision makers better understand various options and make wiser

decisions. The payoff matrix constructed in this paper can provide corresponding recommendations for the agricultural wholesale markets to build e-commerce platforms under different circumstances, which has theoretical guidance and can help the agricultural wholesale markets to make better choices.

2.Secondly, theoretical comparisons can be used to guide practical decision-making in many cases. In fact, theoretical models or theoretical comparisons are often used to guide decision-making in the decision-making process, in order to accurately locate various feasible options and make decisions based on rationality rather than guesswork. Therefore, although theoretical comparisons may not be perfect, they still provide valuable guidance and decision support, which can help people better achieve their goals.

3.The payoff matrix is only a part of the paper, and in the end, we also connected it with reality to prove the problems of insufficient funds[5], incomplete policies[2], and difficult-to-change purchasing habits of wholesalers in the process of building e-commerce platforms for agricultural wholesale markets[3].

R2.9 "In practice, the existence and form of government subsidies are not key factors in establishing e-commerce platforms for agricultural wholesale markets."

Our answer: In the modified manuscript, we discuss the two methods of establishing e-commerce platforms in agricultural wholesale markets: building their own platforms or using third-party platforms. When agricultural wholesale markets have a strong initial inclination to build their own e-commerce platforms, we consider the role of government subsidies in promoting agricultural wholesale markets' self-built e-commerce platforms. We also analyze how agricultural wholesale markets can encourage wholesalers to use their self-built platforms. When agricultural wholesale markets have a strong inclination to use third-party e-commerce platforms, we consider the role of government subsidies in promoting their use.The modifications in the original text are as follows.

Agricultural wholesale markets and wholesalers' behavior choices are somewhat synchronized. When agricultural wholesale markets choose to build their own e-commerce platforms, they can gather information about the specific requirements of wholesalers through face-to-face interactions and online research. By utilizing data analysis tools to analyze wholesalers' behavioral data and behavior trajectories, agricultural wholesale markets can gain insights into wholesalers' search and purchase preferences and identify any problems they may encounter when using the platforms. This insight can help agricultural wholesale markets better understand the needs of wholesalers who are willing to use self-built e-commerce platforms in agricultural wholesale markets which can promote the use of such platforms by wholesalers. Moreover, agricultural wholesale markets can customize their independent and self-built e-commerce platforms according to the needs and preferences of wholesalers. This allows them to provide better user experiences and enhance their competitiveness in the market,and also makes it easier for wholesalers to browse and purchase products, enjoypersonalized services, and obtain accurate product information, thereby enhancing wholesalers' satisfaction and generating additional revenue. When agricultural wholesale markets choose to use third-party platforms, they can actively participate in various activities organized by the platforms to create a tight marketing atmosphere with other merchants. This can promote the use of the platforms by wholesalers who are willing to use third-party e-commerce platforms.
(Line 744-763)

R2.10 "All formulas are not numbered."

Our answer: In the revised article, we have added numbering to all formulas.

R2.11 "What is the basis for parameter assignment in Analysis of the strategy?"

Our answer: We have added the data sources in the paper.The parameter assignments are as follows $b = 30; C_l = 45; C_t = 35; w_2 = 20; I = 30; T_1 = 15; T_2 = 10; A = 30; U_p = 5; u = 0.5; m = 0.5$The data assignments are based on the following.

a.Reference [6]
Yao XG. Government Interference Consumer Purchase Behavior and Brand Construction of Agricultural Products Based on Tripartite Evolutionary Game Theory. Journal of Agro-Forestry Economics and Management. 2023;22(171-181).

b.Based on the real situation: Building e-commerce platforms by agricultural wholesale markets themselves requires more costs compared to using third-party platforms, so we assume$Cl>Ct$.

c.The parameters are simulated using MATLAB software, and the stable points of the system are found to be (0, 1, 0) and (1, 1, 1), which is consistent with the theoretical analysis. Therefore, the parameter assignments in this paper are considered reasonable. The basic policy results for the parameters are shown in the figure below.

Special thanks to you for your good comments.

Best regards,

Yours sincerely

Prof.Qianwen Luo

School of Business, Beijing Wuzi University, Beijing ,China

E-mail:luoqwy@163.com

Telephone:+86-1381-070-8532

References

[1] Sara Encarnação, Fernando P Santos, Francisco C Santos, Vered Blass, Jorge M Pacheco, and Juval Portugali. Paths to the adoption of electric vehicles: An evolutionary game theoretical approach. *Transportation Research Part B: Methodological*, 113:24–33, 2018.

[2] Baotong Liu, Hua Zou, Hao Qin, Huimin Ji, and Yongquan Guo. An evolutionary game analysis of digital transformation of multiagents in digital innovation ecosystems. *Plos one*, 18(7):e0289011, 2023.

[3] Miroslaw Moroz. Acceleration of digital transformation as a result of launching programs financed from public funds: Assessment of the implementation of the operational program digital poland. *Foundations of Management*, 10(1):59–74, 2018.

[4] Ling Zeng Xiaoling Li Xinjian Li, Yang hong. The evolutionary game of three parties participating in the promotion of regional brands of agricultural products. *Chinese Journal of Management Science*, 30(196-209), 2022.

[5] Shijian Wu Xinmin Liu, Xiangyan Sun. Evolution of carbon emission game under dual governance system:analysis from the perspctive of intial wiiingness differentiation. *Systems Engineering*, 37:31–47, 2019.

[6] Xiangyu GuoJiangnan Yao. Government interference consumer purchase behavior and brand construction of agricultural products based on tripartite evolutionary game theory. *Journal of Agro-Forestry Economics and Management*, 22(171-181), 2023.
