# Supplementary material for: Strategies selection for building e-commerce platforms for agricultural wholesale markets: A tripartite evolutionary game perspective
Source: PLoS One. 2024 Jan 25;19(1):e0297360. doi: 10.1371/journal.pone.0297360 (PMC10810498; doi:10.1371/journal.pone.0297360)
Supplement: Attachment — Submitted filename: Response to Reviewers.pdf [file pone.0297360.s003.pdf]

December 19, 2023

Dear Editor and Reviewers,

Thank you for your letter and the reviewers' comments concerning our manuscript entitled "Strategies selection for building e-commerce platforms for agricultural wholesale markets :A tripartite evolutionary game perspective" (ID: PONE-D-23-31750). Those comments are all valuable and very helpful for revising and improving our paper, as well as the important guiding significance to our researches. We have studied comments carefully and have made correction which we hope meet with approval. Below we explain how reviewers' comments were addressed,for easing the reviewing work,the blue text in the following passage is a quotation from the modified original text.

Review 1

R1.1 "The content is supplemented according to the article and method suggestions provided by the reviewers. After summarizing the shortcomings of previous studies, the theoretical framework and model building process of this paper are introduced, and the timeliness and novelty of this paper are compared. You ignored my comments. The authors ignored a lot of the literature in my previous comments, which is very disrespectful. If the authors can't solve this problem, I have to reject the manuscript.

Please explore this issue. In the revised paper, compared with the following literatures, what is the novelty of this study? See the following: "Incentive Mechanism for the Development of Rural New Energy Industry: New Energy Enterprise–Village Collective Linkages considering the Quantum Entanglement and Benefit Relationship", International Journal of Energy Research. https://doi.org/10.1155/2023/1675858. A stochastic differential game of low carbon technology sharing in collaborative innovation system of superior enterprises and inferior enterprises under uncertain environment, https://doi.org/10.1515/math-2018-0056 Please integrate the paper and cite the corresponding literature."

> **Our answer:** We apologize for not fully understanding your comments during the first revision. In the first revision, we made changes only to the title without comparing it to the existing research. Therefore, we have carefully re-read the journal you recommended in the first review and compared it with our paper. We have also integrated the relevant literature into the revised version. First of all, we have cited the relevant literature, emphasizing that digital transformation and upgrading have become an inevitable trend in the era and have had significant impacts in various fields.
>
> The modifications made in the original text are as follows:
> With the rapid development of digital technology, every industry is undergoing transformation and upgrading. In the agricultural field, digital innovation is one of the core elements of agricultural high-end equipment manufacturing system digitization and decarbonization strategy. The application of digital technology can help agricultural enterprises achieve more efficient equipment manufacturing and reduce carbon emissions[16].In the construction industry, the development of digital technology contributes to improving the digital innovation performance of enterprises. The digital integration of the construction-integrated supply chain is a crucial factor affecting the digital innovation of construction industry enterprises[3].In the manufacturing industry, the degree of application of digital technology and the intensity of investment in digital technology by enterprises are crucial factors in the digital transformation of man-

ufacturing enterprises[17]. Through digital innovation, manufacturing enterprises can improve their competitiveness and profit levels and enhance their production processes and management efficiency by effectively utilizing resources, increasing production efficiency, and reducing costs[2].This will lead to higher profits and a more stable market position[17].Digital technology is also impacting agricultural wholesale markets, which are beginning to undergo e-commerce transformation. E-commerce platforms provide a channel of information exchange for all potential traders[13]. With the widespread use of digital technology, many wholesale markets have taken advantage of digital technology, establishing their own commodity trading platforms[19].(Line 42-61)

Literature 1.Incentive Mechanism for the Development of Rural New Energy Industry: New Energy Enterprise–Village Collective Linkages considering the Quantum Entanglement and Benefit Relationship", International Journal of Energy Research. https://doi.org/10.1155/2023/1675858.

Literature 2.A stochastic differential game of low carbon technology sharing in collaborative innovation system of superior enterprises and inferior enterprises under uncertain environment, https://doi.org/10.1515/math-2018-0056

These two papers have provided us with significant inspiration for our article. The first paper adopts the approach of quantum game theory. In recent years, game theory has gradually been combined with quantum information science, where quantum information is primarily used in the game process. The decision-making process of participants in the game is represented by operations on quantum information, and the final strategy outcomes are determined by the final state of the quantum information. The second paper employs the method of stochastic differential game theory. The decision-making process of decision-makers is influenced by various random factors, and the emergence of these random factors leads to significant uncertainty in equilibrium outcomes. Therefore, in recent years, many scholars have adopted the approach of stochastic differential game theory. These methods have provided us with great inspiration for our paper. However, evolutionary game theory emphasizes dynamic equilibrium, which can demonstrate the dynamic evolutionary process of a group over time, enabling the search for optimal strategies and addressing the issue of the imperfect rationality of actors. The main issue we aim to explore in this paper is how to construct e-commerce platforms for agricultural wholesale markets scientifically. We attempt to find the optimal strategies for agricultural wholesale markets under different circumstances; hence, we adopt the approach of evolutionary game theory in this study. We have also restructured the paper and provided a more detailed introduction to game theory in the methodology section for the reader's better understanding.

The modifications made in the original text are as follows:
In recent years, the development of game theory has led to its wide application in various research fields. Traditional game theory assumes that decision-makers are "completely rational." Still, in reality, decision-makers are limited by their own cognition and environmental information differences and can only make decisions that conform to expectations rather than being optimal [1]. Therefore, game theory has gradually combined with other disciplines, enriching and developing game theory. The combination of game theory and quantum information science has formed quantum game theory. Yu et al. applied quantum game theory in the development of rural new energy, explaining the entanglement mechanism between new energy enterprises and rural collectives and establishing incentive mechanisms [18]. The decision-making process of decision-makers is often influenced by various stochastic interference factors, leading to significant uncertainties in the equilibrium results. Therefore, some scholars use the differential game theory to explore the issues. Yin et al. applied differential

game theory to analyze the issue of sharing low-carbon technology between dominant and inferior companies [15]. In the game process, the behavior adjustment of game participants should be regarded as a dynamic evolution process, which is the core where evolutionary game theory breaks the assumption of complete rationality [10]. Evolutionary game theory emphasizes dynamic equilibrium, which can demonstrate a group's dynamic evolution process over time, thus finding optimal strategies and resolving the issue of actors' incomplete rationality [14]. It has been widely applied in supply chain management and system structural evolution research [12]. Tang et al. proposed that when companies conduct e-commerce transformation and upgrading, they need to ensure their own profits and good business operations by constructing an evolutionary game model among companies, government, and digital technology platforms [11]. Dong et al. used an evolutionary game model to discover that the government's subsidy intensity affects the digital transformation process of enterprises and put forward suggestions such as strengthening digital infrastructure construction, establishing a subsidy dynamic adjustment mechanism, and constructing a collaborative and synergetic digital ecosystem [4]. Hou et al. also believed that excessive government intervention would affect market operations [7].(Line 178-207)

At the same time, Literature [7] has also provided us with inspiration and made our paper more comprehensive. Originally, we only discussed the significant role of government subsidies in the digital transformation of enterprises. However, Literature [7] also elucidates the importance of government subsidies being appropriate, which has made our paper more complete.

The modifications made in the original text are as follows:
Hou et al. also believed that excessive government intervention would affect market operations [7](Line 206-207).

Special thanks to you for your good comments.

Review 2

R2.1 "For review comments 6, the meaning of "A" is not fully explained."

Our answer: We have redefined the concept of A, stating that local government can effectively promote the e-commerce transformation process of agricultural wholesale markets through subsidy policies. The central government, in turn, will encourage and support the efforts of local government through incentives. In this way, the central government can also achieve balanced development nationwide, promote economic prosperity in different regions, and strengthen the interaction between local and central government. Therefore, the central government will actively encourage this behavior. Based on the analysis and references mentioned above, if local government chooses to provide subsidies, they will be rewarded by the central government.

The modifications made in the original text are as follows:
Local government can promote the digital transformation process of agricultural wholesale markets through subsidies. The central government recognizes the importance of these measures and encourages and supports the efforts of local government through rewards. Through this mechanism, the central government aims to achieve balanced development nationwide, promote economic prosperity in different regions, and strengthen cooperation and interaction between local and central government. Therefore, if the local government chooses to provide subsidies, it will receive rewards from the central government, referred to as A[9](Line 295-302)

R2.2 "Using the principle of system dynamics to make decisions based on the income matrix can best be combined with the following policy recommendations."

Our answer: In the final stage of policy recommendations, we have integrated current policies and analyzed the situation when wholesalers choose to use either self-built e-commerce platforms or third-party e-commerce platforms in agricultural wholesale markets. Under the current circumstances, the government strongly supports eligible enterprises in establishing e-commerce platforms. In 2022, the Notification from the State Council of China on the 14th Five-Year Plan for the Development of the Digital Economy specifically mentions supporting eligible large enterprises in building integrated e-commerce platforms. In light of this, we have provided corresponding strategies and recommendations. For agricultural wholesale markets that lack the conditions to establish their own e-commerce platforms, they can also use third-party e-commerce platforms, which is also a direction of policy support. The Notification on Several Measures to Promote the High-quality Development of E-commerce by the People's Government of Shandong Province, the People's Republic of China, also emphasized the encouragement of traditional commercial enterprises to expand their online sales share by building self-built platforms or relying on third-party platforms. Therefore, if enterprises choose to use third-party e-commerce platforms, we have also provided corresponding strategies and recommendations to promote the e-commerce transformation process of agricultural wholesale markets through various means.

The modifications made in the original text are as follows.
As an important policy maker, the government should take proactive measures to promote the development of the agricultural wholesale markets through e-commerce. In recent years, the trend of third-party e-commerce platforms' monopoly has become increasingly apparent. These platforms not only charge high usage fees and commissions but also impose restrictions on wholesalers. This situation has prompted eligible companies to transition to the self-built e-commerce platforms gradually. In addition, the "14th Five-Year Plan for Digital Economy Development" issued by the State Council in 2022 also mentioned supporting large enterprises with certain conditions to establish comprehensive e-commerce platforms. Therefore, if enterprises have a strong interest in building their own e-commerce platforms, the government can provide funding through fiscal budget arrangements to subsidize the construction of agricultural wholesale markets that choose to build their own e-commerce platforms to encourage and support the establishment of these platforms. In addition, the government can lower the cost of building self-built e-commerce platforms for agricultural wholesale markets through measures such as monetary supply and interest rate regulation, such as lowering loan interest rates, to further promote the construction of these platforms. The "Notice on Promoting High-Quality Development of E-commerce Measures" issued by the Shandong Provincial People's Government also emphasizes encouraging traditional commercial distribution enterprises to expand their network sales share through self-built or third-party platforms. When the agricultural wholesale markets choose to use third-party e-commerce platforms, the government can directly subsidize the platform service fees that these markets pay for maintenance, technical support, and promotion. At the same time, the government can help agricultural wholesale markets reduce the resources and human resources required for using third-party e-commerce platforms, as well as the expenses of managing and operating an e-commerce business, by providing low-interest loans and other methods(Line 708-823).

R2.3 "According to review comments 11, when using data for simulation verification, the relationship between data is difficult to be convincing."

Our answer: We obtained data from four sources. The first source is government

policies. The second source is literature. The third source is real-world situations. The fourth source is expert opinions and materials. Meanwhile, through simulating the basic situation, we found that the system's stable points existed at (0,1,0) and (1,1,1). This is consistent with the theoretical analysis and further validates the accuracy of our assignments.

The modifications made in the original text are as follows.
This article obtains the initial values of various parameters through four methods. The first method is from policies. Since the issuance of the "Opinions on Accelerating the Development of the Circulation Industry" by the State Administration for Industry and Commerce of China in 2013, policies have been introduced across the country to support the development of e-commerce in agricultural wholesale markets after the establishment of online sales platforms. In Foshan, Guangdong, the government provides incentives of 0.4 million and 0.2 million yuan respectively for the construction of five-star and four-star smart agricultural wholesale markets. In Dongguan, Guangdong, the government provides a subsidy of 30% of the investment for the construction of smart agricultural wholesale markets, with a maximum of 0.5 million yuan. In Shishi, Fujian, and Haining, Zhejiang, the local finance department provides subsidies of up to 0.1 million yuan for the transformation of agricultural wholesale markets into e-commerce. In Qingdao, Shandong, the municipal government provides an incentive of up to 0.3 million yuan for the transformation into e-commerce of agricultural wholesale markets. In Jiaozuo, Henan, and Huaihua, Hunan, the municipal government provides financial support of up to 0.5 million yuan for the construction of smart agricultural wholesale markets. The implementation of these policies has stimulated the transformation of agricultural wholesale markets into e-commerce. Based on policies across the country, it is assumed that $I = 30$ (Units: ten thousand yuan). The second method is based on existing literature. Referring to the research by Cui et al. [8]on platform enterprises, government, and consumers, $T_1 = 15$ and $T_2 = 10$ (Units: ten thousand yuan)are assumed. Referring to the research by Li et al. [9]on the promotion of regional public brands for agricultural products and the analysis by Guo et al.[6] on the construction of agricultural product brands, $A = 30$ and $U_p = 5$(Units: ten thousand yuan) are assumed. $B = 30$ (Units: ten thousand yuan) is assumed based on the research by Guo et al. [6]on the construction of agricultural product brands, and $W_2 = 20$ (Units: ten thousand yuan) is assumed based on the research by Sara et al. [5] on the adoption of electric vehicles and the research by Guo et al. [6]on the construction of agricultural product brands. The third method is based on field research. This article assumes that the incentives from the government for agricultural wholesale markets to build their own e-commerce platforms and use third-party platforms are the same at the initial stage, that is, u=0.5 and m=0.5. At the same time, the influence of different levels of government support on the behavior of agricultural wholesale markets and wholesalers is also discussed in the subsequent discussions. The fourth method is based on interviews with managers of agricultural wholesale markets and data collection. This article obtained that the cost for agricultural wholesale markets to choose to build their own e-commerce platforms is approximately 0.4 million to 0.5 million yuan through interviews with managers of agricultural wholesale markets. By reviewing the fees for joining third-party platforms, it is found that the fees mainly include platform service fees, margin, promotion fees, and technical service fees.The platform service fees are about 0.05 million to 0.2 million yuan, the margin is about 0.05 million to 0.1 million yuan, the promotion fees are about 0.03 million to 0.06 million yuan, and the technical service fees, which are commission, are about 5% to 12% of the sales revenue. Therefore, it is assumed that $C_l = 45$ and $C_t = 35$(Units: ten thousand yuan). Based on the above analysis, the assigned values for each parameter are as follows: $b = 30; C_l = 45; C_t = 35; w_2 = 20; I = 30; T_1 = 15; T_2 = 10; A = 30; U_p = 5; u = 0.5; m = 0.5$(Units: ten thousand yuan).(Line 394-442)

Special thanks to you for your good comments.

Best regards,

Yours sincerely

Prof.Qianwen Luo

School of Business, Beijing Wuzi University, Beijing ,China

E-mail:luoqwy@163.com

Telephone:+86-1381-070-8532

References

[1] Charles Crawford and Catherine Salmon. *Evolutionary psychology, public policy and personal decisions.* Psychology Press, 2004.

[2] Tong Dong, Shi Yin, and Nan Zhang. New energy-driven construction industry: Digital green innovation investment project selection of photovoltaic building materials enterprises using an integrated fuzzy decision approach. *Systems*, 11(1):11, 2022.

[3] Tong Dong, Shi Yin, and Nan Zhang. The interaction mechanism and dynamic evolution of digital green innovation in the integrated green building supply chain. *Systems*, 11(3):122, 2023.

[4] Zhiyong Dong and Peichao Dai. Dynamic mechanism of digital transformation in equipment manufacturing enterprises based on evolutionary game theory: Evidence from china. *Systems*, 11(10):493, 2023.

[5] Sara Encarnação, Fernando P Santos, Francisco C Santos, Vered Blass, Jorge M Pacheco, and Juval Portugali. Paths to the adoption of electric vehicles: An evolutionary game theoretical approach. *Transportation Research Part B: Methodological*, 113:24–33, 2018.

[6] Xiangyu Guo and Jiangnan Yao. Government interference consumer purchase behavior and brand construction of agricultural products based on tripartite evolutionary game theory. *Journal of Agro-Forestry Economics and Management*, 22(171-181), 2023.

[7] Xiaona Hou, Xiangxiao Gao, Shi Yin, and Jianmin Li. A three evolutionary game model for driving mechanism of industry-university-research collaborative innovation in agricultural innovation ecosystems. *Plos one*, 18(8):e0289408, 2023.

[8] Cui Li, Hong Li, and Changqi Tao. Evolutionary game of platform enterprises, government and consumers in the context of digital economy. *Journal of Business Research*, 167:113858, 2023.

[9] Xinjian Li, Hong Yang, Ling Zeng, and Xiaoling Li. The evolutionary game of three parties participating in the promotion of regional brands of agricultural products. *Chinese Journal of Management Science*, 30(196-209), 2022.

[10] Martin A Nowak and Karl Sigmund. Evolutionary dynamics of biological games. *science*, 303(5659):793–799, 2004.

[11] Decai Tang, Jiannan Li, Shaojian Qu, and Valentina Boamah. Tripartite collaboration among government, digital technology platform, and manufacturing enterprises: Evolutionary game model. *Sustainability*, 15(10):7946, 2023.

[12] Jun Tanimoto. *Fundamentals of evolutionary game theory and its applications*. Springer, 2015.

[13] Tobias Wulfert and Erik Karger. A bibliometric analysis of platform research in e-commerce: Past, present, and future research agenda. *Corp. Ownersh. Control*, 20:185–200, 2022.

[14] YY Yi and HM Liu. Evolutionary game theory in economic research. *J. Bus. Econ*, 8:8–13, 2005.

[15] Shi Yin and Baizhou Li. A stochastic differential game of low carbon technology sharing in collaborative innovation system of superior enterprises and inferior enterprises under uncertain environment. *Open Mathematics*, 16(1):607–622, 2018.

[16] Shi Yin, Yuexia Wang, and Junfeng Xu. Developing a conceptual partner matching framework for digital green innovation of agricultural high-end equipment manufacturing system toward agriculture 5.0: A novel niche field model combined with fuzzy vikor. *Frontiers in Psychology*, 13:924109, 2022.

[17] Shi Yin, Nan Zhang, Kifayat Ullah, and Shuo Gao. Enhancing digital innovation for the sustainable transformation of manufacturing industry: a pressure-state-response system framework to perceptions of digital green innovation and its performance for green and intelligent manufacturing. *Systems*, 10(3):72, 2022.

[18] Yuanyuan Yu and Shi Yin. Incentive mechanism for the development of rural new energy industry: New energy enterprise–village collective linkages considering the quantum entanglement and benefit relationship. *International Journal of Energy Research*, 2023, 2023.

[19] Wen Zhang, Ting Hou, and Qinglong Gou. Choosing a self-built or an intermediary platform for hosting winner-take-all crowdsourcing contests? *Annals of Operations Research*, pages 1–22, 2023.
